# Supplementary figures and images for: Bioinformatic analysis linking genomic defects to chemosensitivity and mechanism of action
Source: PLoS One. 2021 Apr 28;16(4):e0243336. doi: 10.1371/journal.pone.0243336 (PMC8081165; doi:10.1371/journal.pone.0243336)

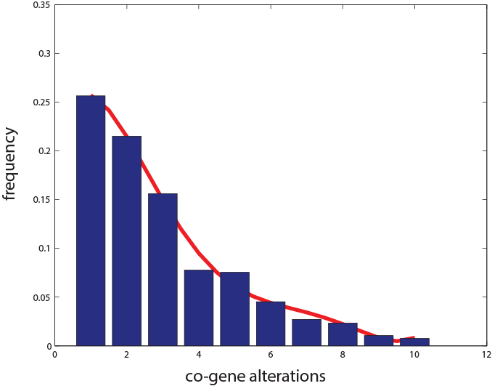

Supplement: S1 Fig — (TIF) [file pone.0243336.s001.tif]

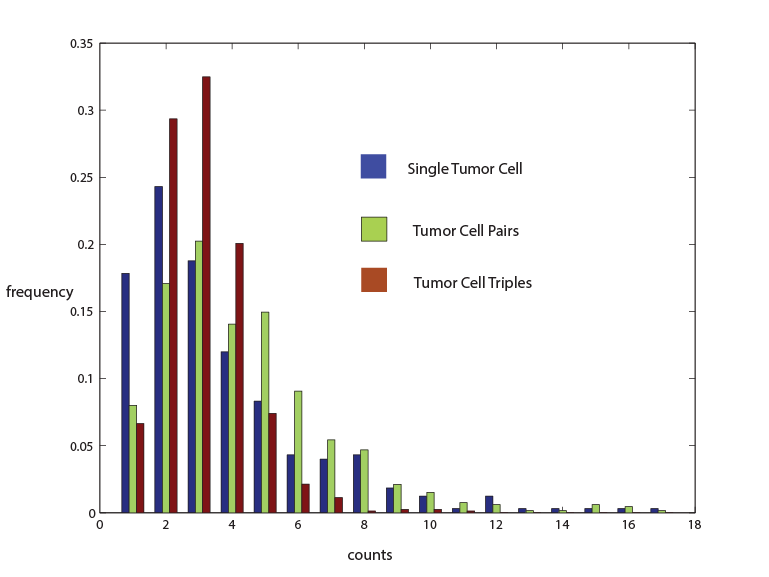

Supplement: S2 Fig — (TIF) [file pone.0243336.s002.tif]

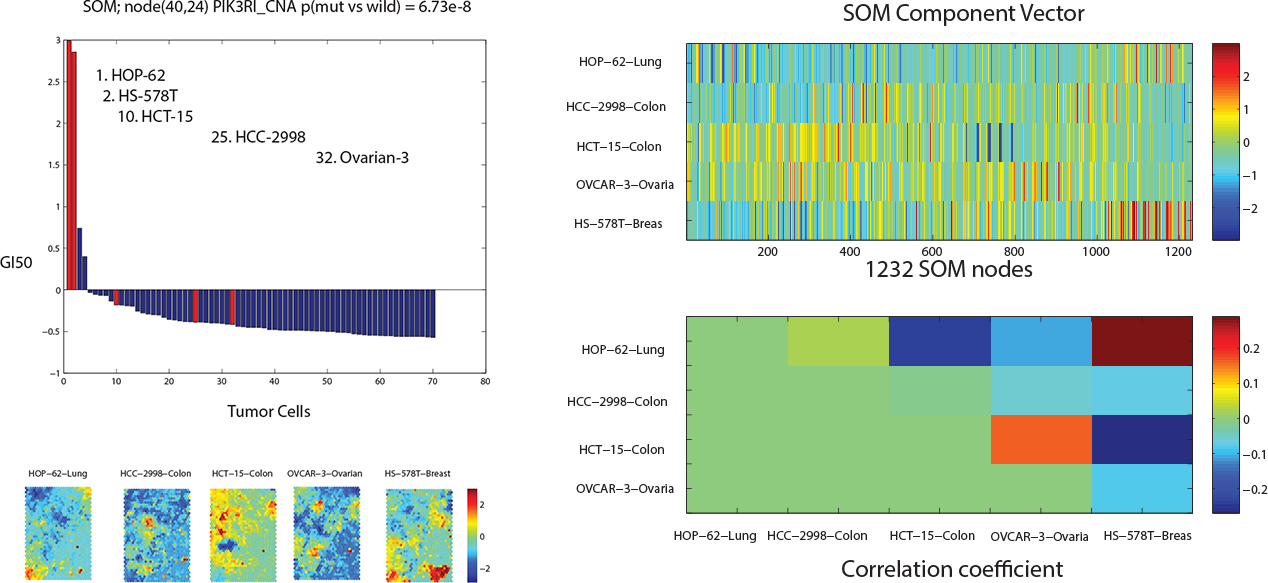

Supplement: S3 Fig — (TIF) [file pone.0243336.s003.tif]

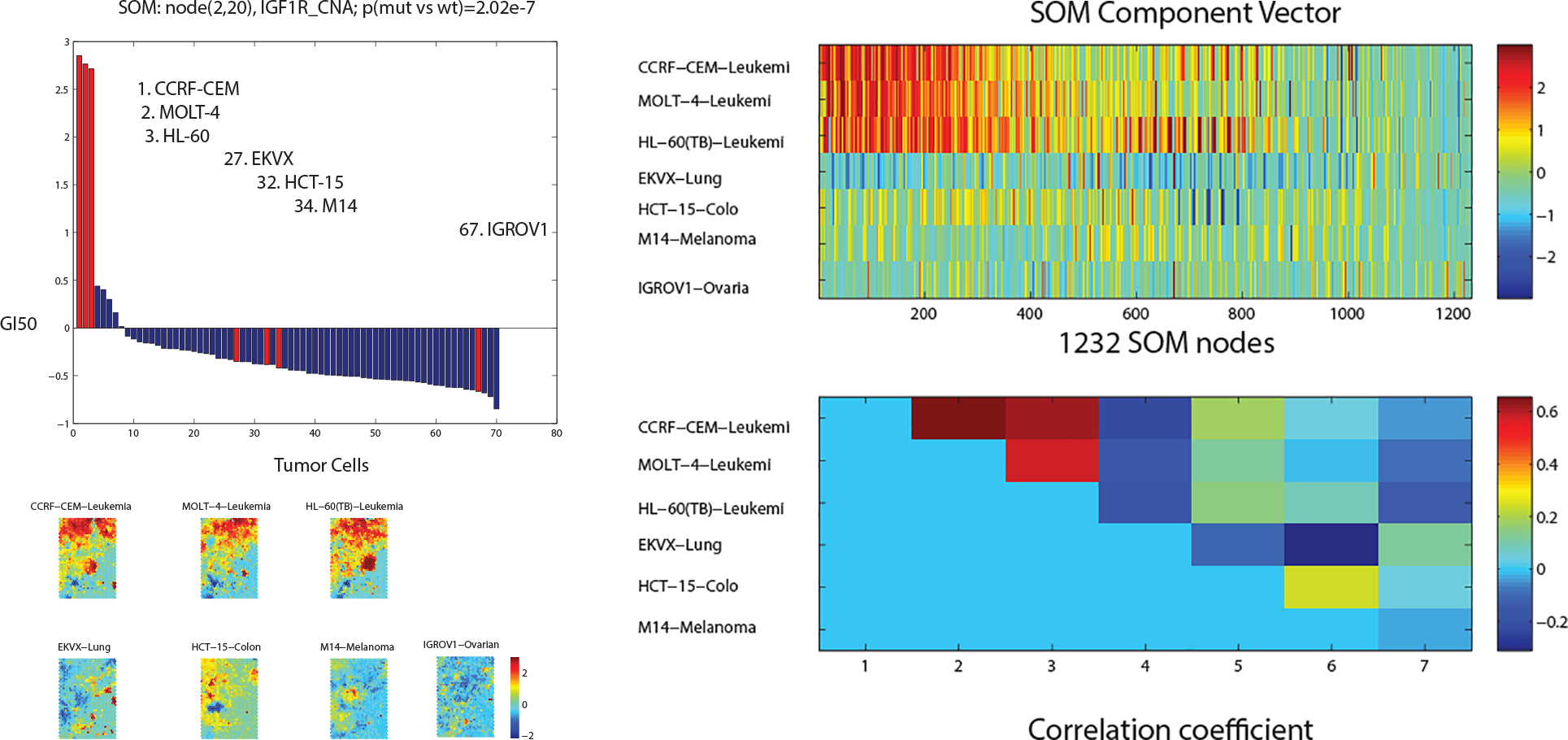

Supplement: S4 Fig — (TIF) [file pone.0243336.s004.tif]
