# Supplementary material for: Bioinformatic analysis linking genomic defects to chemosensitivity and mechanism of action
Source: PLoS One. 2021 Apr 28;16(4):e0243336. doi: 10.1371/journal.pone.0243336 (PMC8081165; doi:10.1371/journal.pone.0243336)
Supplement: S1 File — (DOCX) [file pone.0243336.s005.docx]

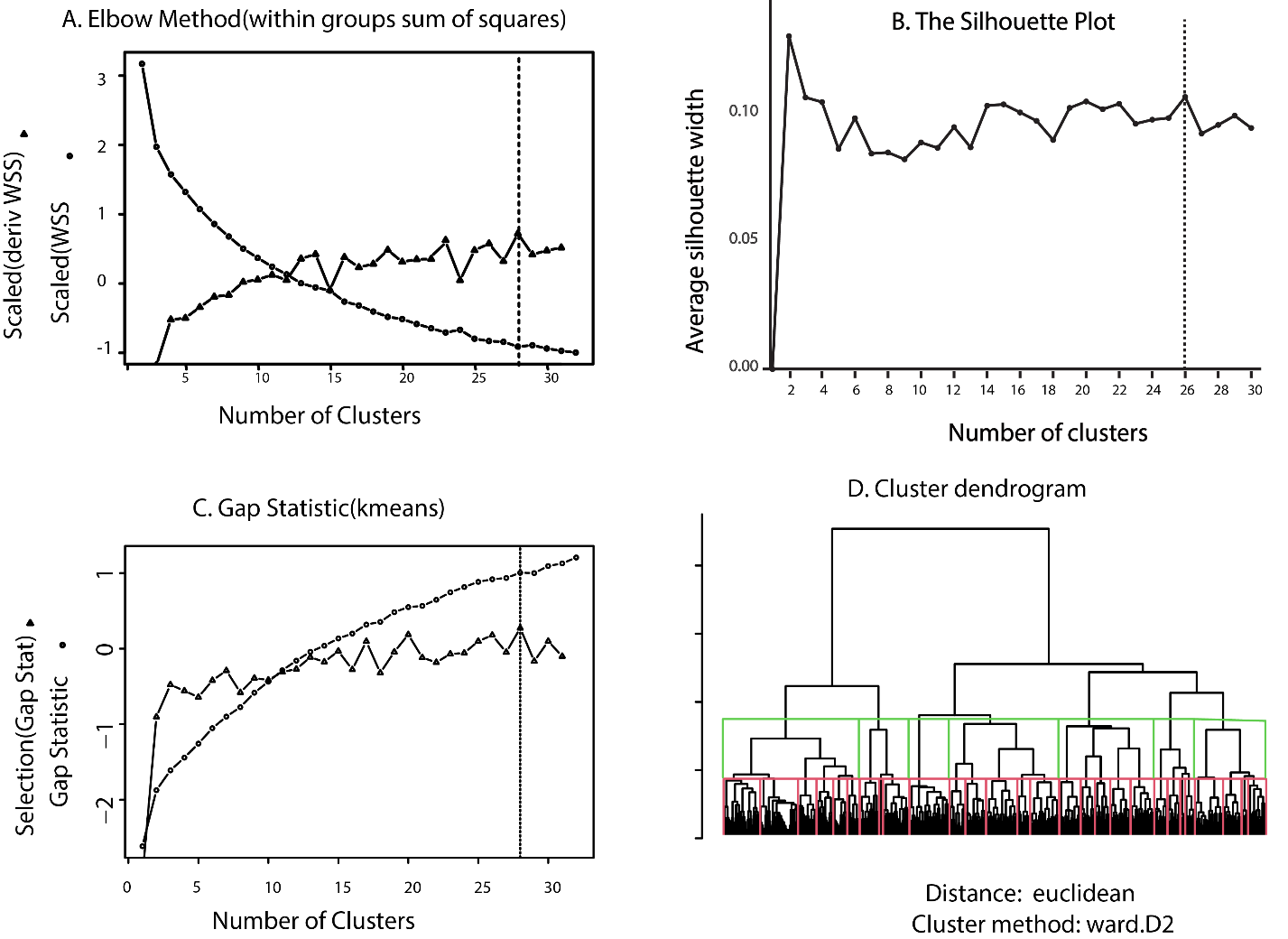


**Figure 1**. Results for selecting optimal cluster size using the **elbow** method (**Panel A**), **silhouette** method (**Panel B**) and the **gap**_**statistic** method (**Panel C**). Selection for the **elbow** method is based on the largest local derivative of the within groups sum of squares (**Panel A** triangles), the maximum silhouette width (**Panel B**) and the first non-negative value for Gap(k)-(Gap(k + 1)−sd_k+1_) (**Panel C** triangles). These results indicate an optimal number of clusters in the 26-28 range. **Panel D** displays the GI50_codebook_ dendrogram (Euclidean, Ward’s) with cuts at 28 (red lines) and 7 clusters (green lines), respectively.


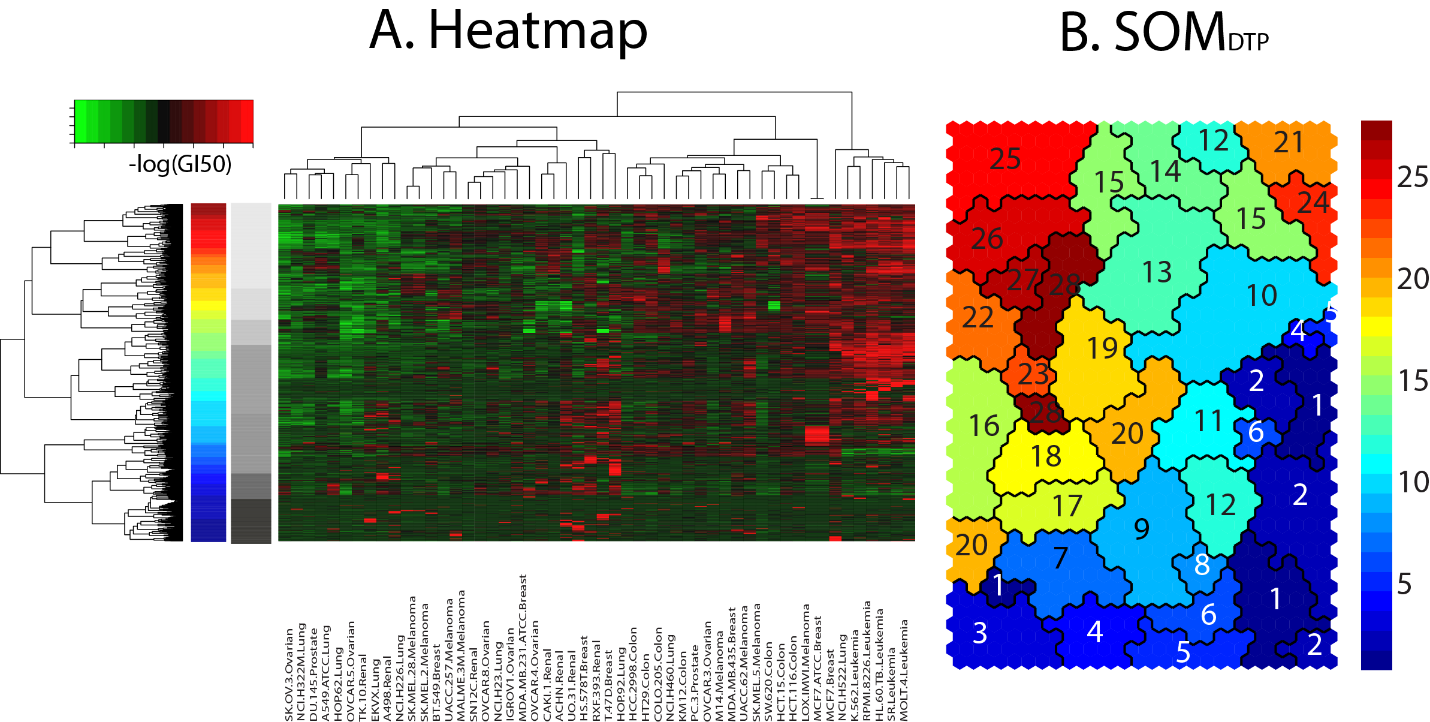


**Figure 2**: **Panel A** displays a heatmap of GI50_codebook_, colored spectrally from green(chemoinsensitive) to red(chemosensitive) response. Dendrogram at the left represents hierarchical clustering (Euclidean, Ward’s) of GI50_codebooks_ (reproduced from **Figure 1 Panel D**). **Panel B** displays the SOM_DTP_ colored according to hierarchical cutree [18] specified at the optimal number of 28 meta-clades. The 28 colors appear spectrally from meta-clade 1 (dark blue), at the bottom of the hierarchical dendrogram, to meta-clade 28 (dark red), at the top of the hierarchical dendrogram. Grayscale bar adjacent to the 28 meta-clade spectrally colored bar displays the 7 meta-clades groupings. The NCI60 tumor cell lines clustered in the heatmap are ordered, left to right, as: SK.OV.3.Ovarian, NCI.H322M.Lung, DU.145.Prostate, A549.ATCC.Lung, HOP.62.Lung, OVCAR.5.Ovarian, TK.10.Renal, EKVX.Lung, A498.Renal, NCI.H226.Lung, SK.MEL.28.Melanoma, SK.MEL.2.Melanoma, BT.549.Breast, UACC.257.Melanoma, MALME.3M.Melanoma, SN12C.Renal, OVCAR.8.Ovarian, NCI.H23.Lung, IGROV1.Ovarian, MDA.MB.231.ATCC.Breast, OVCAR.4.Ovarian, CAKI.1.Renal, ACHN.Renal, UO.31.Renal, HS.578T.Breast, RXF.393.Renal, T.47D.Breast, HOP.92.Lung, HCC.2998.Colon, HT29.Colon, COLO.205.Colon, NCI.H460.Lung, KM12.Colon, PC.3.Prostate, OVCAR.3.Ovarian, M14.Melanoma, MDA.MB.435.Breast, UACC.62.Melanoma, SK.MEL.5.Melanoma, SW.620.Colon, HCT.15.Colon, HCT.116.Colon, LOX.IMVI.Melanoma, MCF7.ATCC.Breast, MCF7.Breast, NCI.H522.Lung, K.562.Leukemia, RPMI.8226.Leukemia, HL.60.TB..Leukemia, SR.Leukemia, MOLT.4.Leukemia, CCRF.CEM.Leukemia


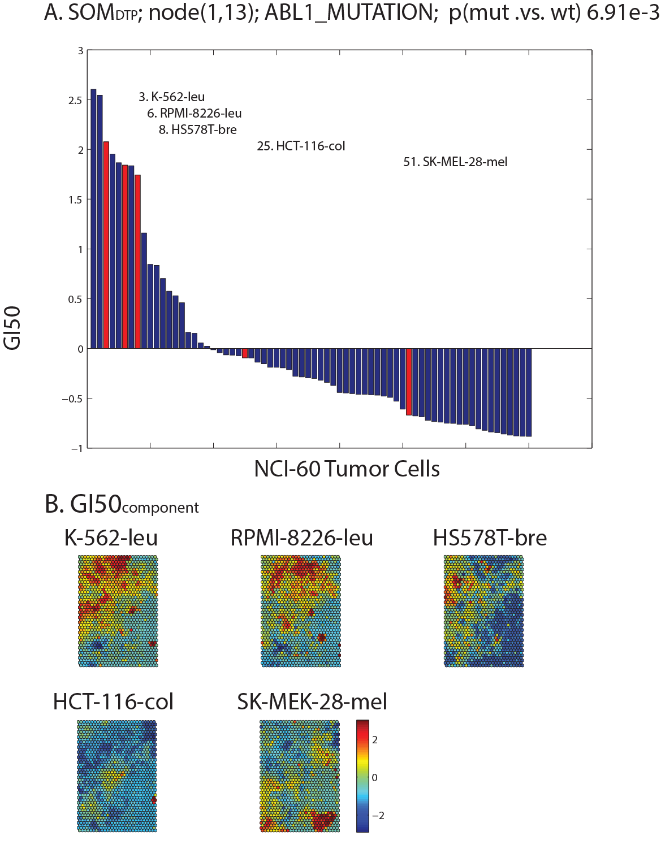


**Figure 3**. **Panel A** displays GI50_codebook_ for SOM_1,13_, ordered from most to least chemosensitivity. The 5 tumor cell lines with the defective *ABL1* gene appear as red bars. **Panel B** displays GI50_component_ for the 5 tumor cell lines with defective *ABL1*. SOM_DTP_ nodes are colored spectrally from highest chemosensitivity (red) to lowest chemosensitivity (blue).


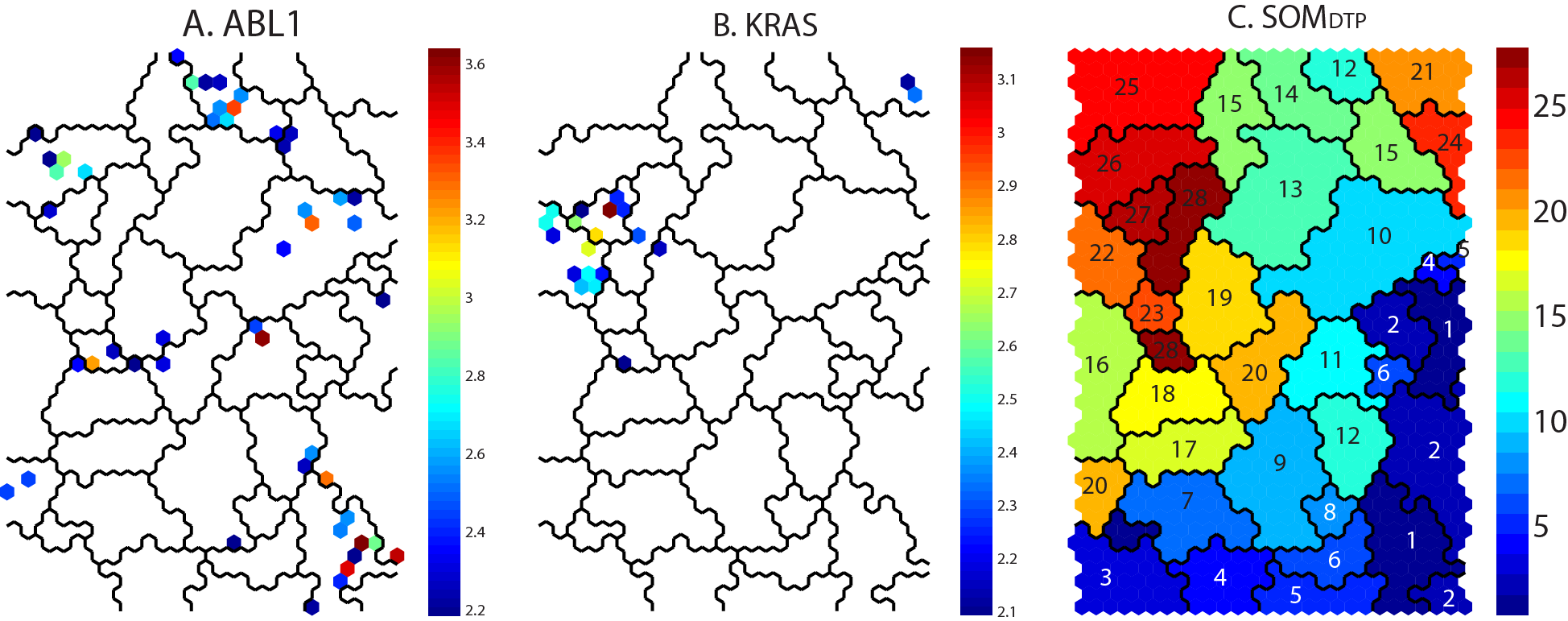


**Figure 4**: **Panels A** and **B** display significant chemosensitive SOM_DTP_ nodes (projected as their t-statistic from a Student’s t-test; blue:least, red:most significant) for tumor cell lines with defective *ABL1* and *KRAS*, respectively. **Panel C** displays the 28 SOM_DTP_ meta-clades.


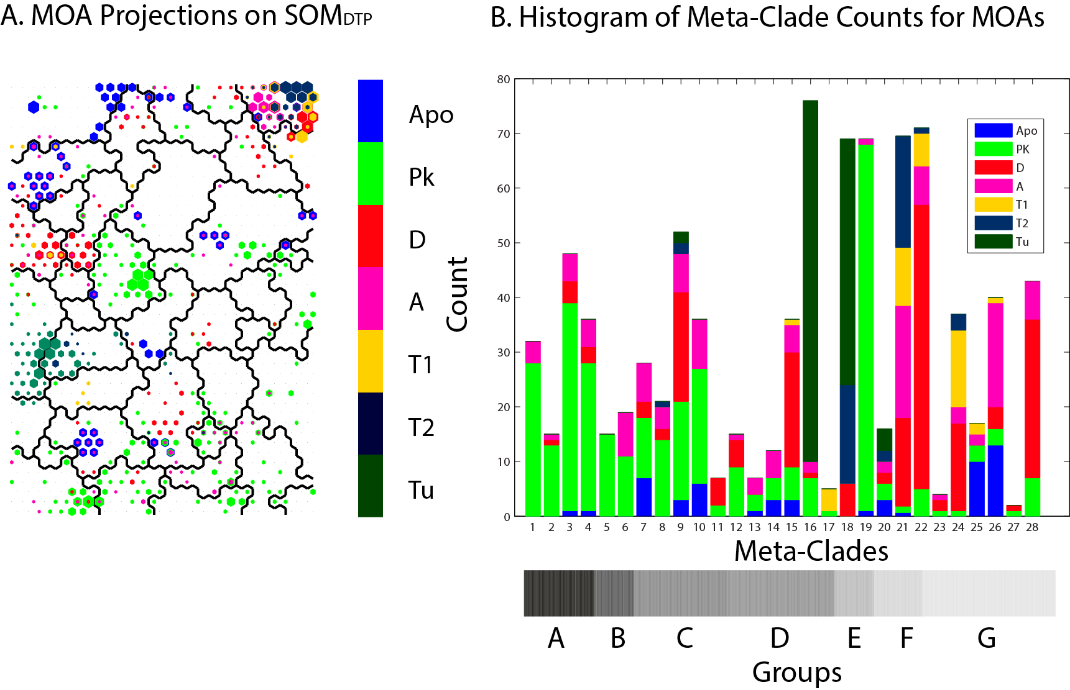


**Figure 5**: **Panel A:** SOM_DTP_ projections for FDA approved compounds for the primary CellMiner assigned MOAs. Projections include the top 10^th^ percentile of SOM_DTP_ nodes for each compound. **Panel B:** histogram of the counts for these primary MOAs across SOM meta-clade groups. Primary MOAs appear color-coded in each vertical bar, with their heights corresponding to MOA counts in each meta-clade. Horizontal grayscale bar below Panel B indicates meta-clade groups **A**:**G** (reproduced from **Figure 2 Panel A**).

**
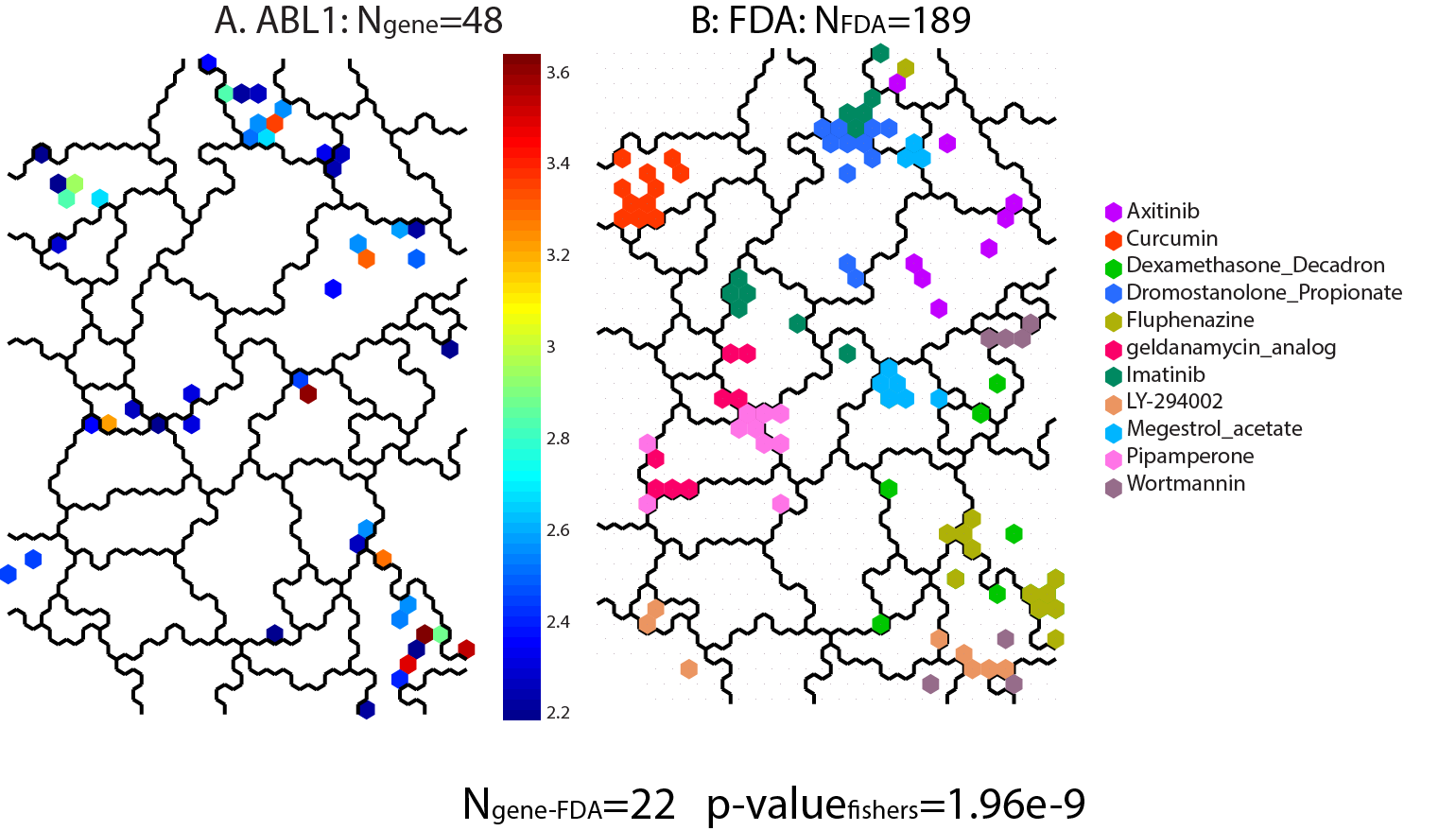
**

**Figure 6: Panel A** displays the significant SOM_DTP_ nodes for *ABL1* (N_gene_=48). Eleven FDA compounds are co-projected to N_gene_; yielding 6 MOAs. The SOM_DTP_ in **Panel B** displays the top 10^th^ percentile of projections for FDA compounds sharing these MOAs (N_FDA_=189). The intersection of N_gene_ and N_FDA_ = 22, yielding a Fishers exact p-value of 1.958262e-09, log(p-value= -20.05).


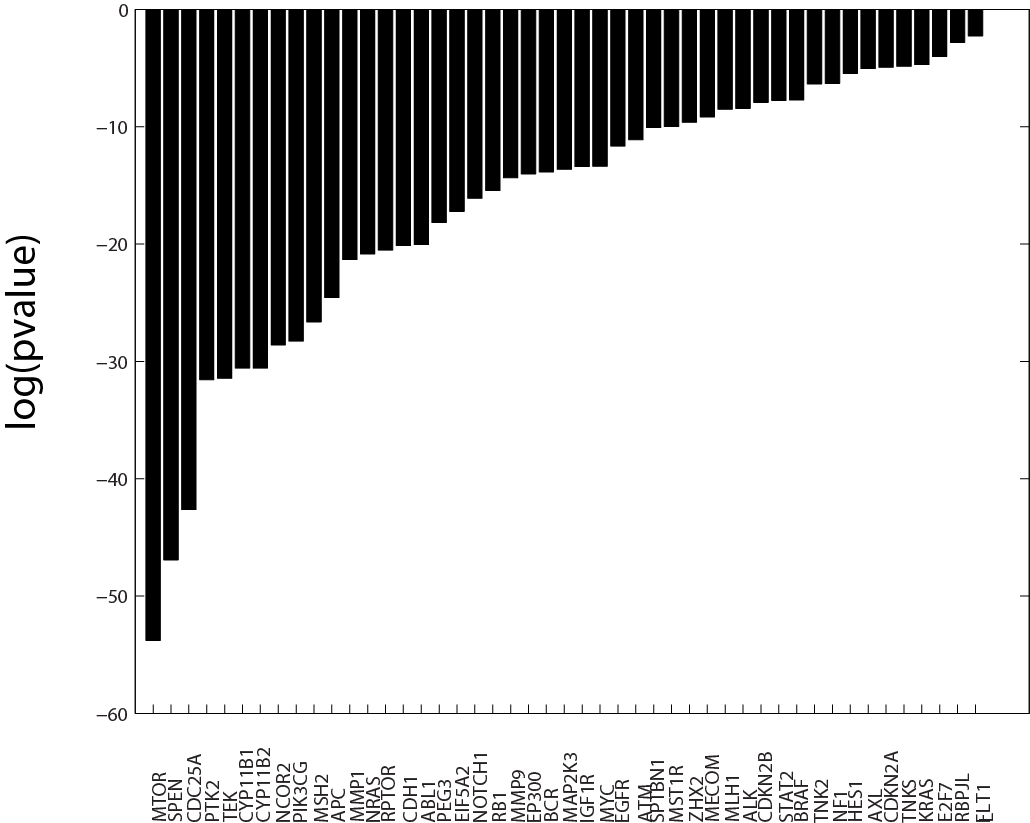


**Figure 7**: Fisher’s exact scores (log(pvalue), pvalue<=0.05). Results are based on classifications using up to the 10^th^ best SOM projection nodes for FDA compounds. Forty-seven defective genes have significant Fisher’s exact scores when tested over the complete SOM_DTP_.


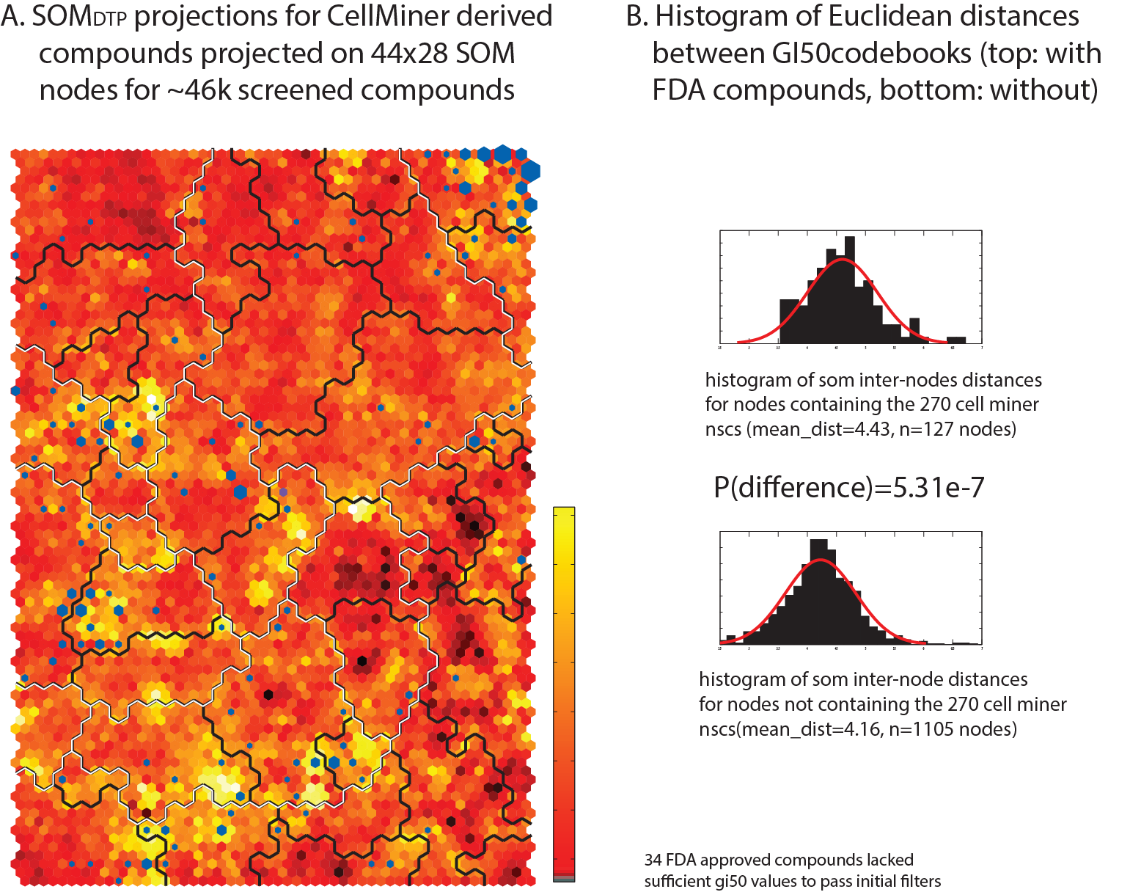


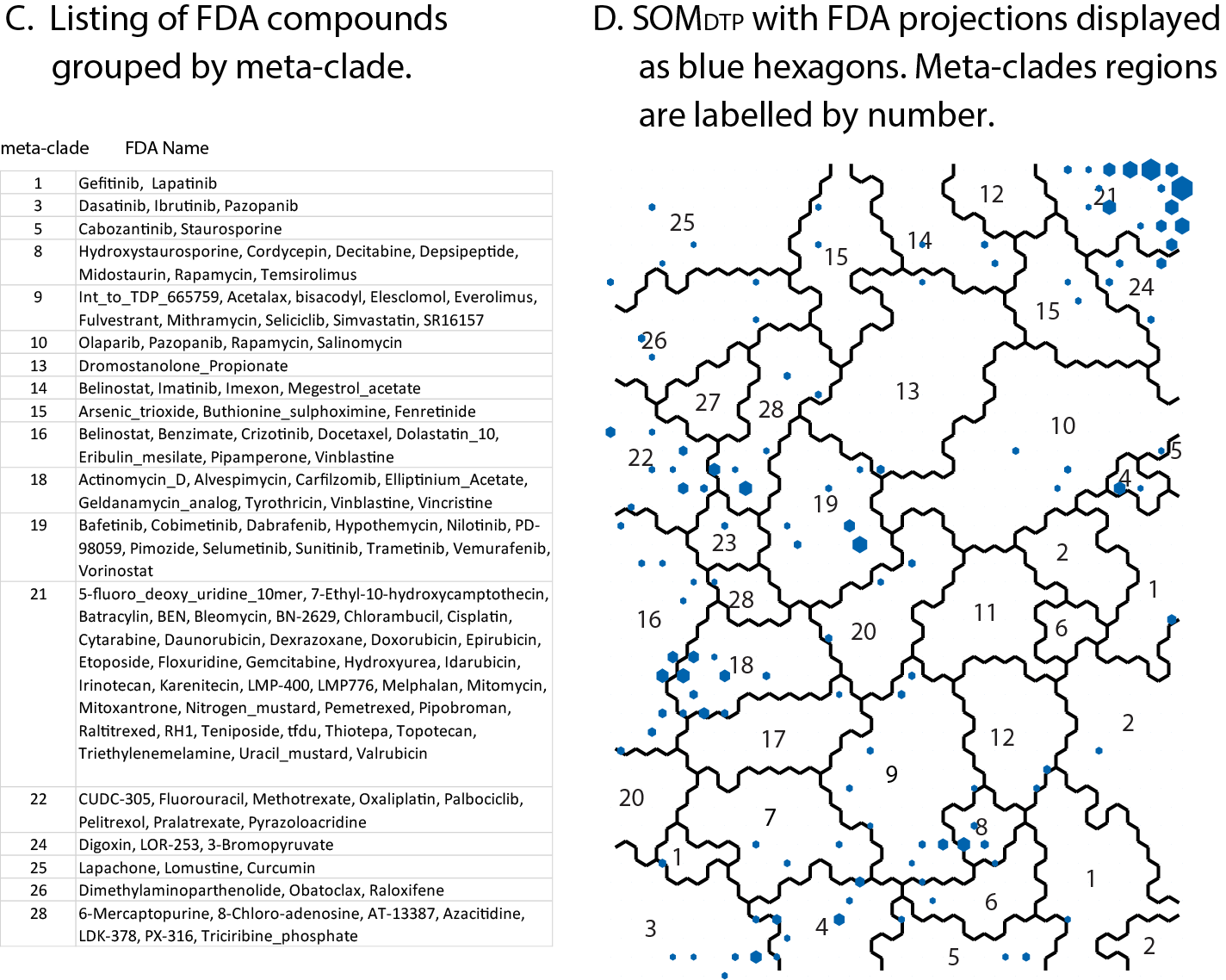


**Figure 8: Panel A.** SOM_DTP_ is colored according to similarity of GI50_codebooks_, where the most similar node neighbors are displayed in deep red and the most dis-similar node neighbors appear in bright yellow (see vertical bar adjacent to SOM_DTP_). The 28 optimal meta-clade boundaries are displayed as a black line, with the boundaries of the 7 meta-clade groups super-imposed as a white line. FDA approved compounds are projected onto SOM_DTP_ as blue hexagons, where hexagons are sized according to the number of FDA agents appearing in any node. **Panel B** displays the between node GI50_codebook_ Euclidean distances for nodes with FDA compound projections (top) and without (bottom). **Panel C** lists FDA compound names grouped by 28 meta-clades. **Panel D** displays SOM_DTP_ with FDA compounds (blue hexagons), meta-clade boundaries (solid lines) and meta-clade labels as numbers. FDA approved projections to SOM_DTP_ nodes are listed in **S5 master_appendix sheet** **appendix_Table_III**.


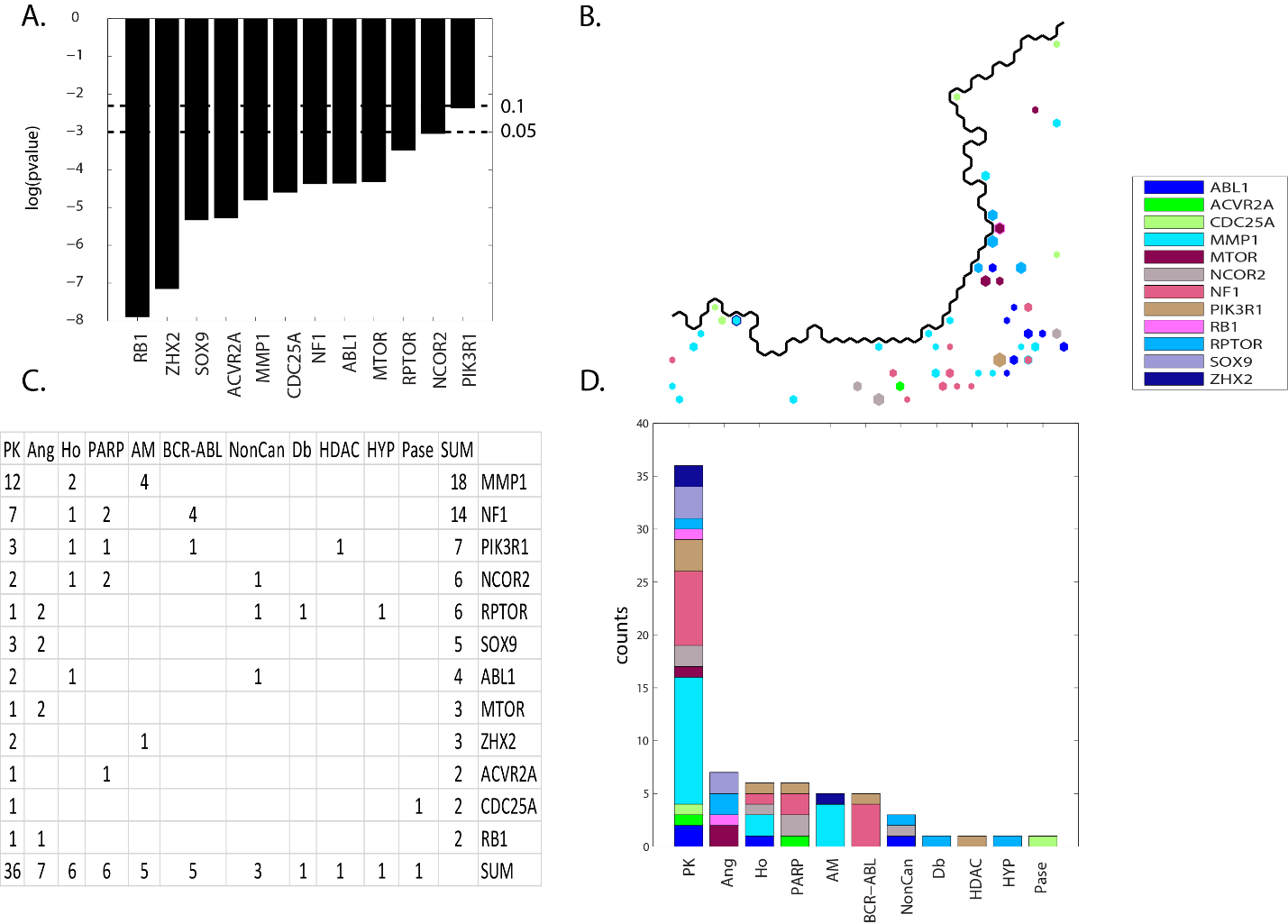


**Figure 9:** **Panel A** displays the contingency scores, ordered left to right, from the most to least significance. The horizontal dashed lines represent significance thresholds of p<=0.05 (lower line) and p<=0.1 (upper line). **Panel** **B** displays the SOM_DTP_ co-projections of significant defective genes and MOAs for FDA compound. Only co-occurrences for SOM_DTP_ projections of FDA compounds are displayed**.** The SOM_DTP_ region displayed in **Panel B** represents the boundary for meta-clades 1 through 6 (see the white border in **Figure 8 Panel A**). **Panel C** lists the counts for co-occurrence (see **S6 master_appendix sheet gp_A**). **Panel D** displays the tabular results in **Panel C** as a histogram. Node colors for defective genes correspond to the legend inserted into the upper left panel. The counts displayed in **Panel C** represent the top 10^th^ percentile of SOM_DTP_ co-projections for FDA compounds. A consistent coloring scheme is used for this and all subsequent figures, such that all defective genes presented in the **RESULTS** are assigned a unique color. **S13 master_appendix_sheet** **gp_A_FDA** list the counts for each FDA and MOA entry for these significant genes.


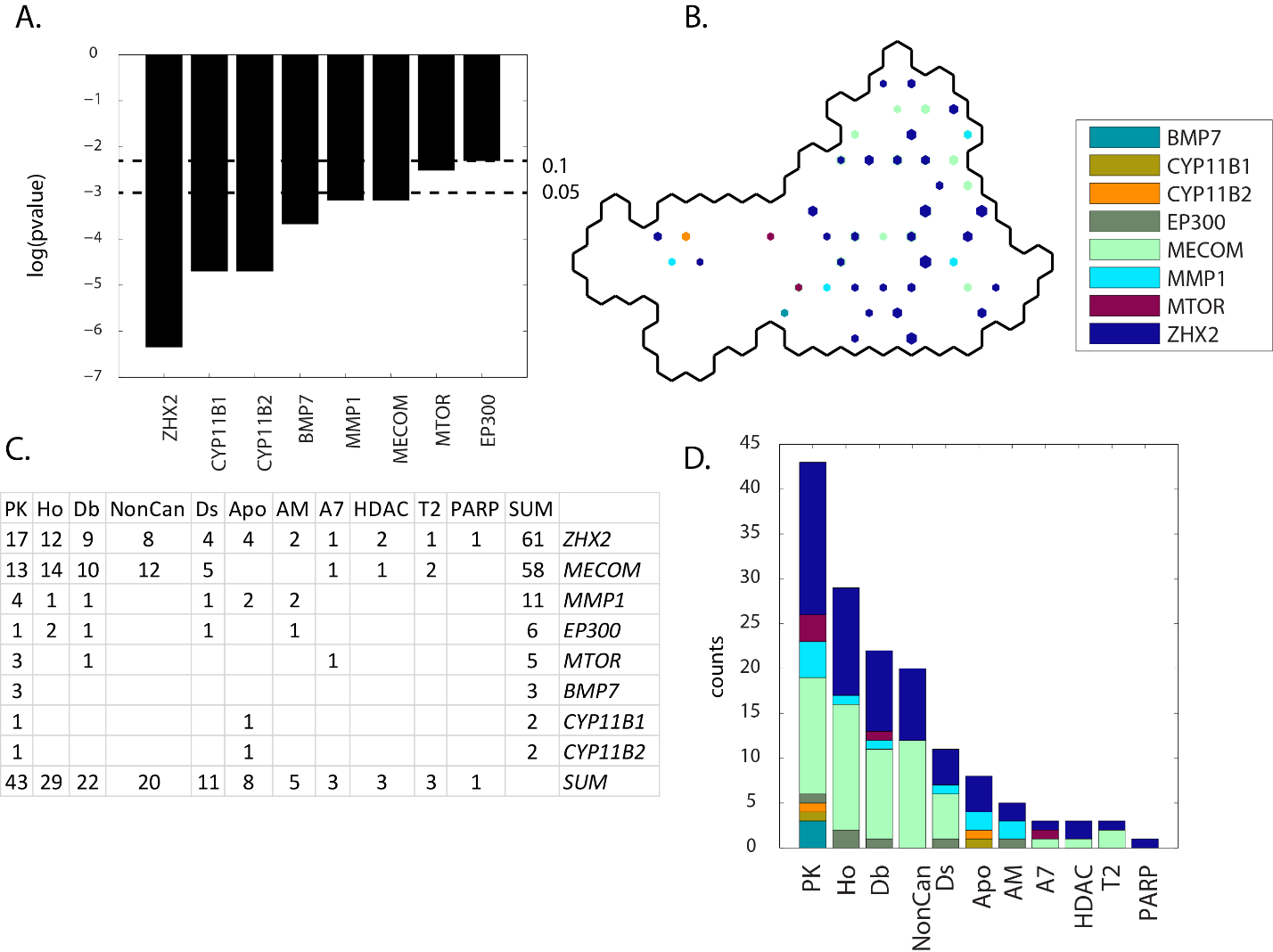


**Figure 10.** Results for group **B**(meta-clades 7 through 9). The SOM_DTP_ region displayed in **Panel B** represents the boundary for meta-clades 7 through 9 (see the white border in **Figure 8 Panel A**). **S7 master_appendix sheet gp_B** lists the table in **Panel C.** See the legend of **Figure 9** for details. **S14 master_appendix sheet gp_B_FDA** lists the FDA compounds associated with these defective genes.


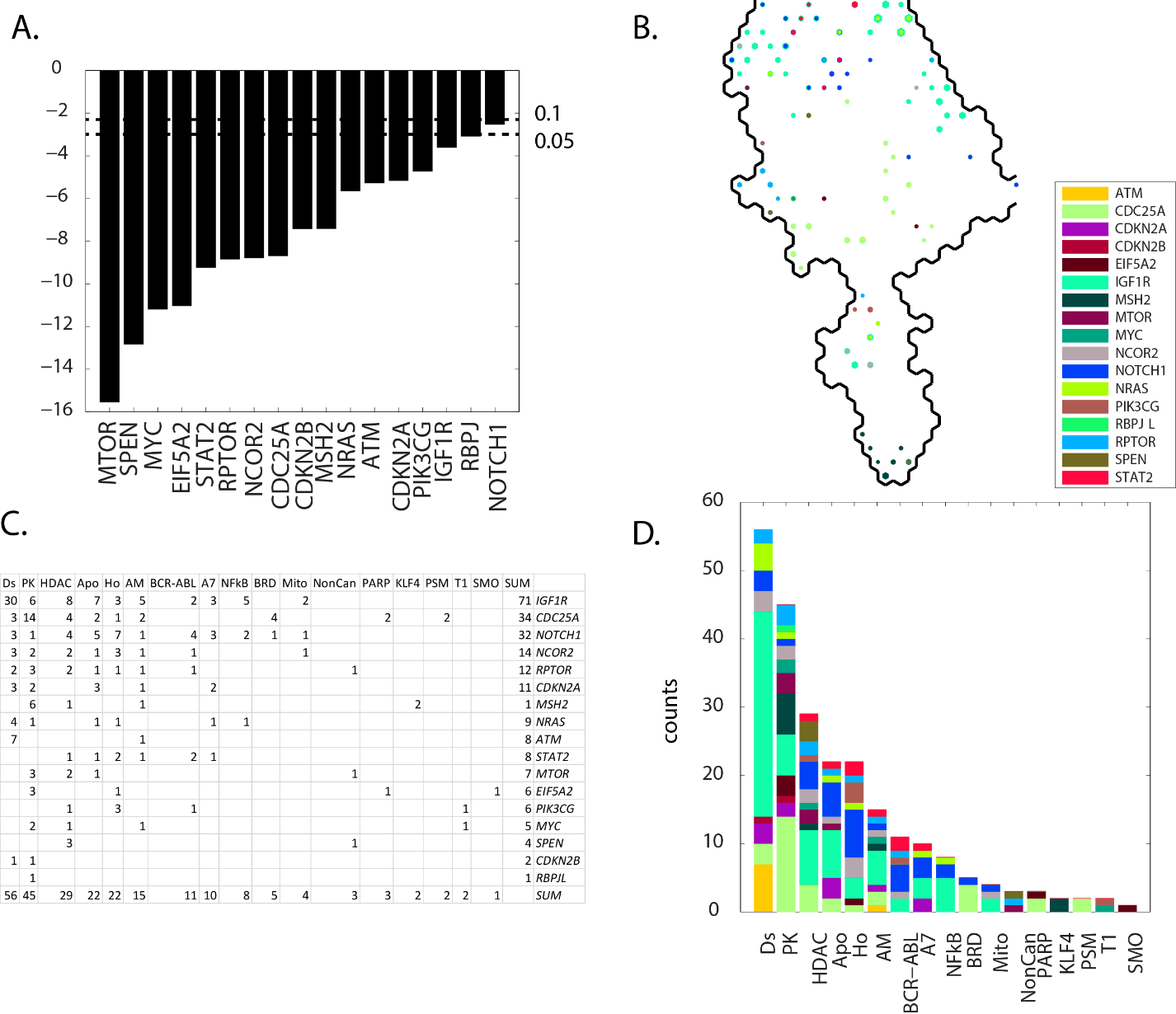


**Figure 11.** Results for group **C**(meta-clades 10 through 15). The SOM_DTP_ region displayed in **Panel B** represents the boundary for meta-clades 10 through 15 (see the white border in **Figure 8 Panel A**). **S8 master_appendix sheet gp_C** lists the table in **Panel C.** See the legend of **Figure 9** for details. **S15 master_appendix sheet gp_C_FDA** lists the FDA compounds associated with these defective genes.


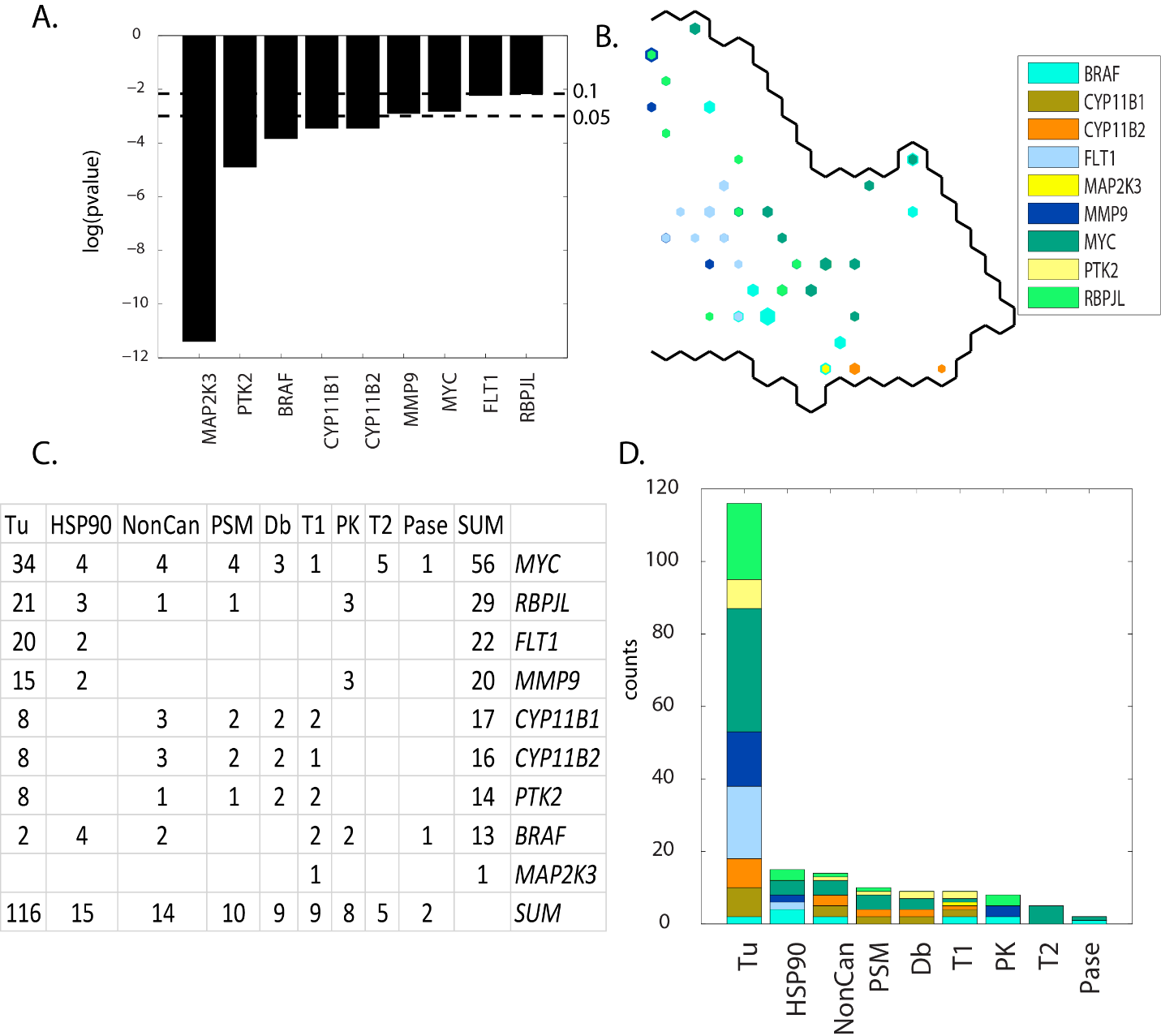


**Figure 12:** Results for group **D**(meta-clades 16 through 18). The SOM_DTP_ region displayed in **Panel B** represents the boundary for meta-clades 16 through 18 (see the white border in **Figure 8 Panel A**). **S9 master_appendix sheet gp_D** lists the table in **Panel C.**  See legend of **Figure 9** for additional details. **S16 master_appendix sheet gp_D_FDA** lists the FDA compounds associated with these defective genes.


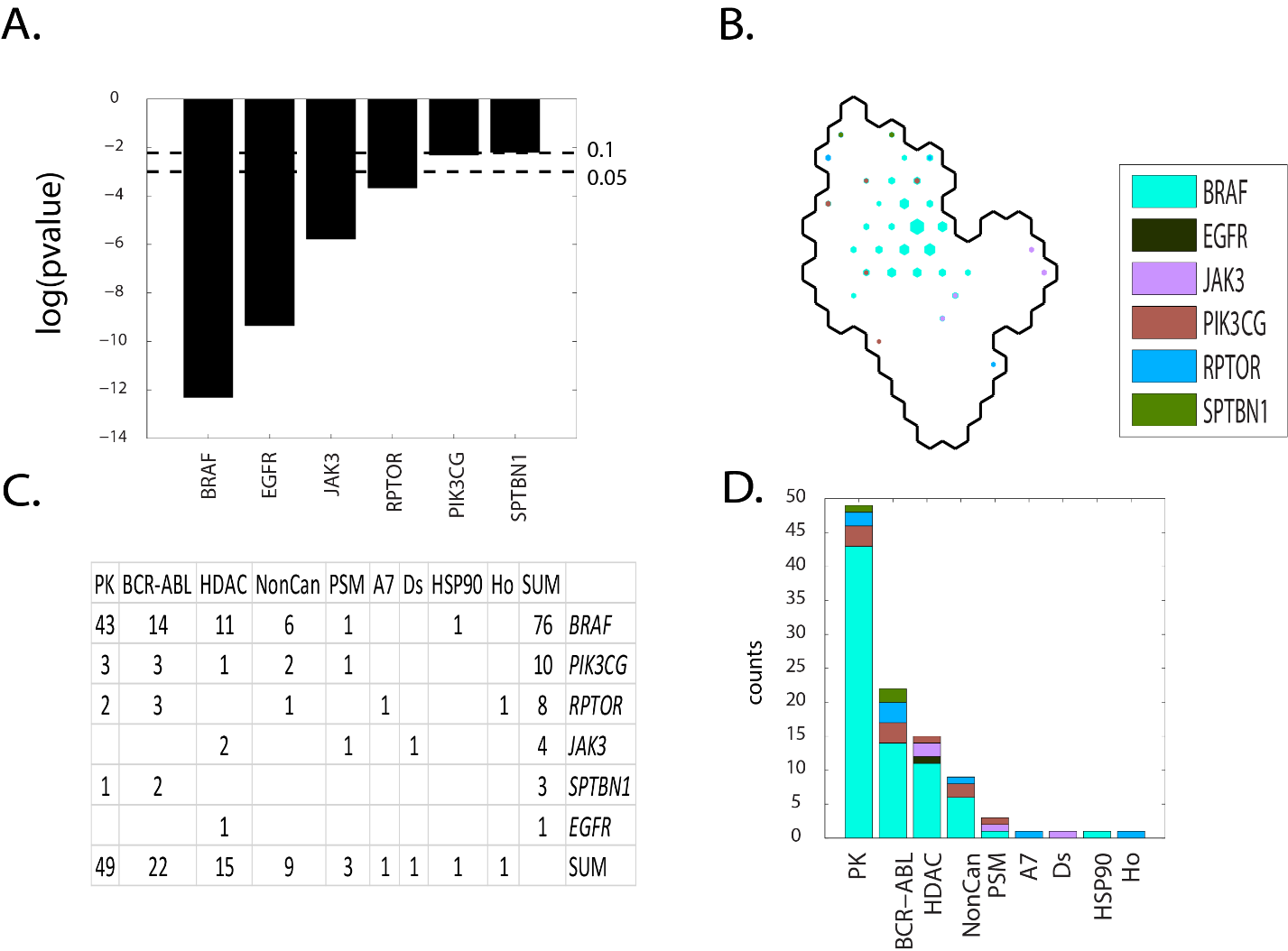


**Figure 13:** Results for group **E**(meta-clades 19 through 20). The SOM_DTP_ region displayed in **Panel B** represents the boundary for meta-clades 19 through 20 (see the white border in **Figure 8 Panel A**). **S10 master_appendix sheet gp_E** lists the table in **Panel C.**  See legend to **Figure 9** for additional details. **S17 master_appendix sheet gp_E_FDA** lists the FDA compounds associated with these defective genes.


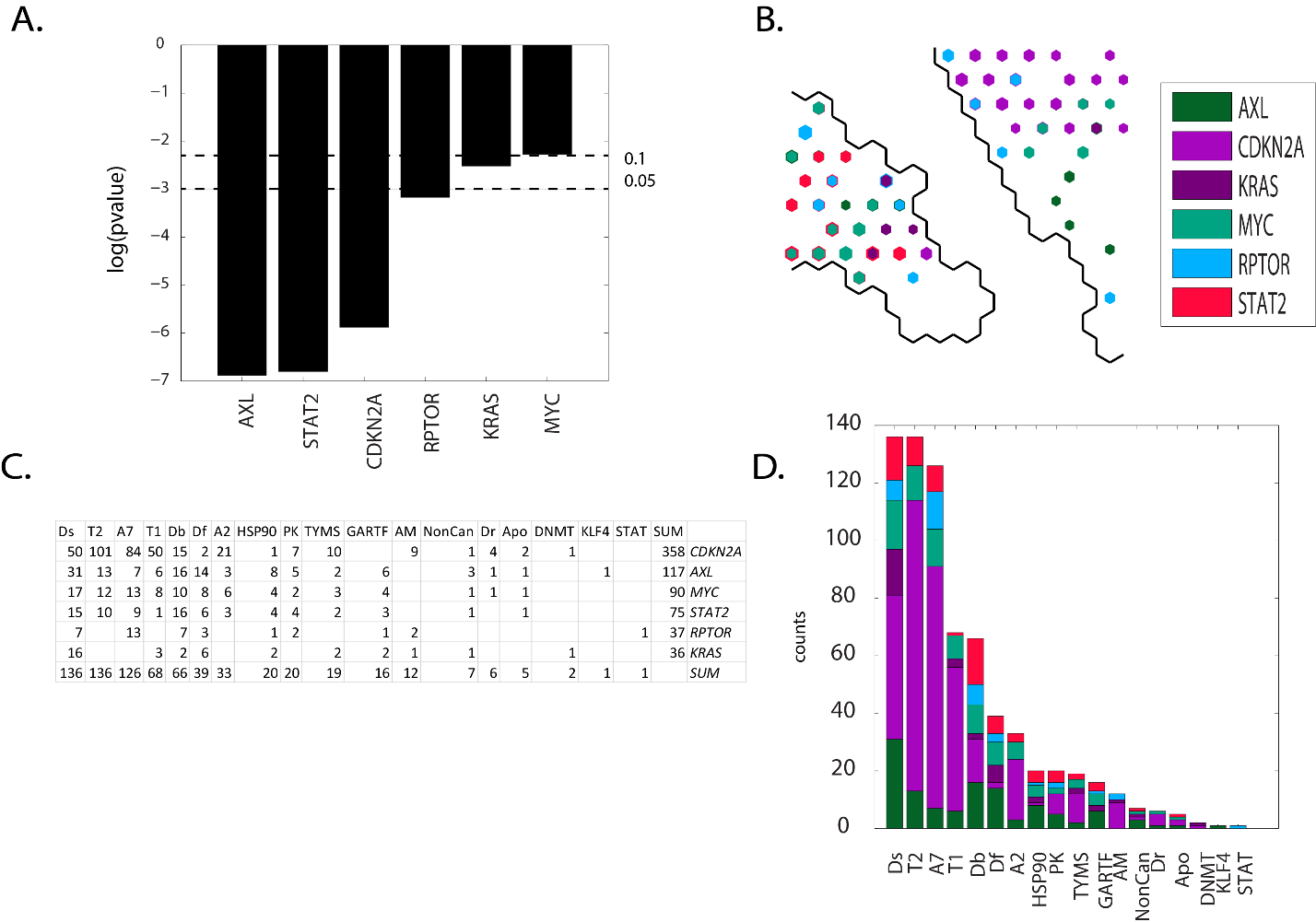


**Figure 14:** Results for group **F**(meta-clades 21 through 24). The SOM_DTP_ region displayed in **Panel B** represents the boundary for meta-clades 21 through 24 (see the white border in **Figure 8 Panel A**). **S11 master_appendix sheet gp_F** lists the table in **Panel C.**  See legend to **Figure 9** for additional details. **S18 master_appendix sheet gp_F_FDA** lists the FDA compounds associated with these defective genes.


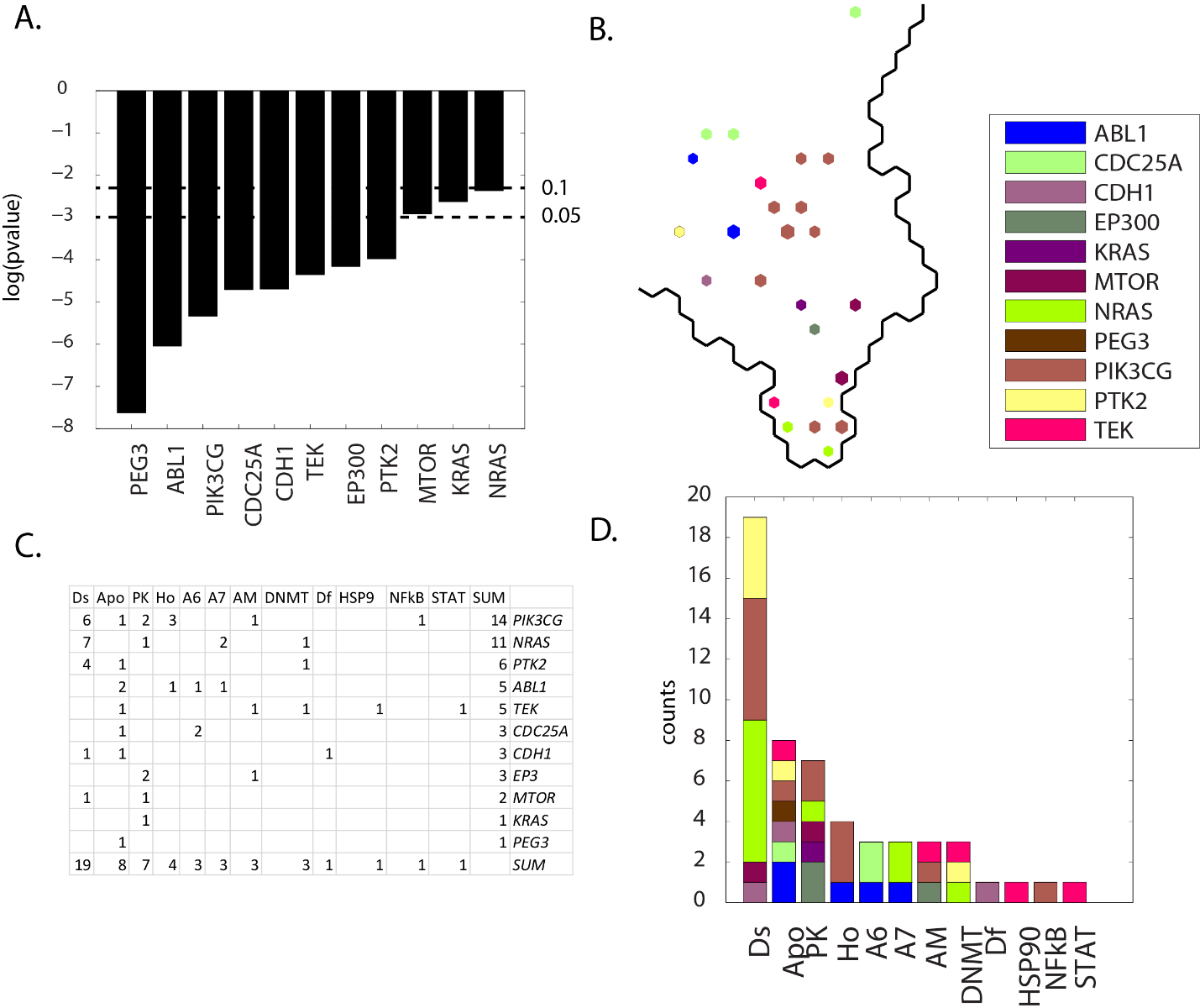


**Figure 15:** Results for group **G**(meta-clades 25 through 28). The SOM_DTP_ region displayed in **Panel B** represents the boundary for meta-clades 25 through 28 (see the white border in **Figure 8 Panel A**). **S12 master_appendix sheet gp_G** lists the table in **Panel C.**  See legend to **Figure 9** for additional details. **S19 master_appendix sheet gp_G_FDA** lists the FDA compounds associated with these defective genes.
