# Supplementary material for: Bioinformatic analysis linking genomic defects to chemosensitivity and mechanism of action
Source: PLoS One. 2021 Apr 28;16(4):e0243336. doi: 10.1371/journal.pone.0243336 (PMC8081165; doi:10.1371/journal.pone.0243336)

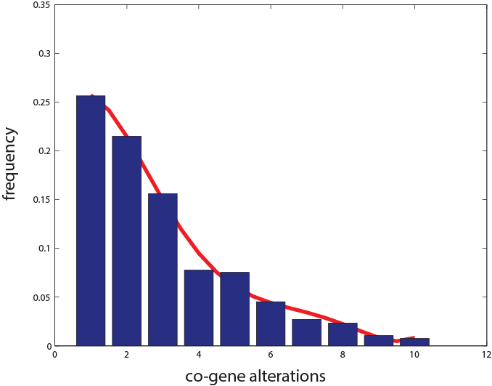


**Appendix Figure 1:** Frequency histogram for numbers of defective genes shared by pairs of tumor cells.

As a further comparison, **Appendix Figure 2** displays the histogram of defective gene frequencies appearing in one(blue), two(green) and three(red) NCI60 tumor cells. This result indicates that co-defective genes comprised of doublets and triplets are more common (higher frequency) compared to the appearance of only one defective gene (consistent with Ikediobi et al.).


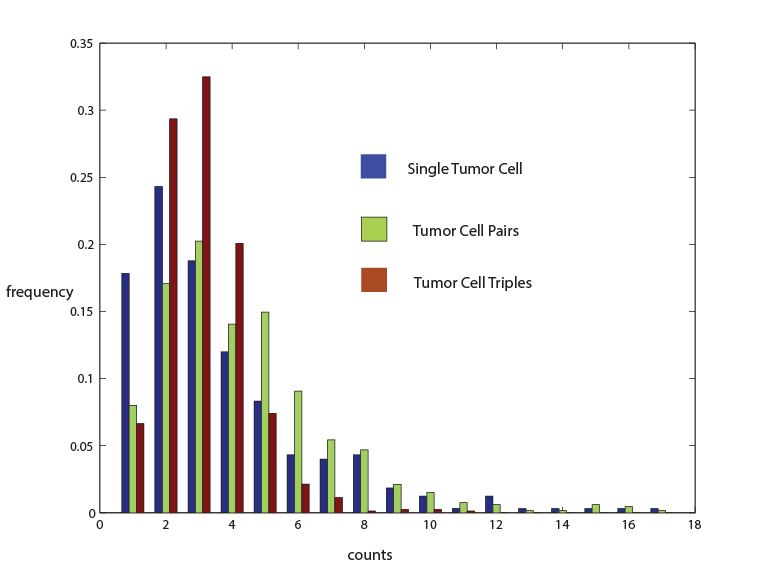


**Appendix Figure 2** displays the frequencies of defective genes based on their mutual appearance in only one tumor cell (blue), pairs of tumor cells (green) and triples of tumor cells (red).

**Appendix Figures 1** and **2** share the features of i) having their highest frequencies for cases of co-defective gene doublets and triplets and ii) exhibiting frequencies > 0.05 for 8 or fewer co-defective genes. Clearly co-defective genes in the NCI60 are not uncommon.

**Appendix Table I** lists the most frequent singlets, doublets and triplets of defective genes observed in the NCI60. **Appendix Table II** lists their counts. Inspection finds CDC25A, TP53, CDKN2A, CDKN2B, MYC, BRAF, EP300, KRAS, NOTCH2 and PTK2 as the top ten most frequently occurring defective genes. To summarize, co-defective gene doublets and triplets find these top ten defective genes appearing in combination with themselves, and other defective genes. This accounting supports an analysis that includes groups of tumor cells according to combinations (i.e.one, two, three, etc.) of defective genes.

**Appendix Table I:**

| # tumor cells | Singlets | # tumor cells | Doublets | # tumor cells | Triplets |
| --- | --- | --- | --- | --- | --- |
| 41 | CDC25A | 15 | CDC25A:CDKN2A | 12 | CDC25A:CDKN2A:CDKN2B |
| 27 | TP53 | 12 | CDC25A:TP53 | 3 | CDC25A:HES4:MMP23B |
| 21 | CDKN2A | 8 | BRAF:CDC25A | 3 | APC:CDC25A:MECOM |
| 18 | CDKN2B | 6 | CDKN2A:CDKN2B | 2 | CDKN2A:CDKN2B:KRAS |
| 14 | MYC | 6 | APC:CDC25A | 2 | CDKN2A:CDKN2B:FLT3 |
| 12 | BRAF | 5 | CDC25A:MYC | 2 | CDC25A:SPTBN1:TP53 |
| 12 | EP300 | 4 | HES4:MMP23B | 2 | CDC25A:SPEN:TP53 |
| 12 | KRAS | 4 | CDC25A:MET | 2 | CDC25A:PTEN:TP53 |
| 12 | NOTCH2 | 3 | SPEN:TP53 | 2 | CDC25A:PEG3:TP53 |
| 11 | PTK2 | 3 | CDC25A:NOTCH1 | 2 | CDC25A:MET:PIK3CG |
| 10 | ATM | 3 | CDC25A:MAP2K3 | 2 | CDC25A:MAPK7:TP53 |
| 10 | MAP2K3 | 3 | CCND1:TP53 | 2 | CDC25A:MAP2K3:TP53 |
| 10 | ROS1 | 3 | ATM:CDC25A | 2 | CDC25A:FLT1:MET |
| 10 | SPEN | 2 | ROS1:STAT2 | 2 | CDC25A:EGFR:TP53 |
| 9 | APC | 2 | PEG3:TP53 | 2 | CDC25A:CDKN2A:MYC |
| 9 | CCND1 | 2 | NOTCH2:TP53 | 2 | BRAF:CDC25A:TP53 |
| 9 | MMP1 | 2 | NF1:TP53 | 2 | BRAF:CDC25A:MAP2K3 |
| 9 | NF1 | 2 | EP300:ERBB2 | 1 | RFNG:RPTOR:SOX9 |
| 9 | TEK | 2 | CDC25A:PEG3 | 1 | PDGFRA:TEK:TP53 |
| 9 | ZHX2 | 2 | CDC25A:PAK4 | 1 | MYC:SPTBN1:TP53 |
| 8 | ALK | 2 | CDC25A:NOTCH3 | 1 | MMP1:PDGFRA:TP53 |
| 8 | CYP11B1 | 2 | CDC25A:MSH2 | 1 | MLH1:RET:RICTOR |
| 8 | CYP11B2 | 2 | CDC25A:MECOM | 1 | MLH1:NOTCH2:TP53 |
| 8 | EGFR | 2 | CDC25A:KRAS | 1 | MAP2K3:MAPK7:TP53 |
| 8 | FLT3 | 2 | CDC25A:JAK2 | 1 | KRAS:MYC:TP53 |
| 8 | KDR | 2 | CDC25A:EP300 | 1 | KRAS:MYC:PTK2 |
| 8 | MECOM | 2 | BMP6:CDC25A | 1 | KDR:ROS1:ZHX2 |
| 8 | NCOR1 | 2 | ALK:CDC25A | 1 | HSD3B1:HSD3B2:NOTCH2 |
| 8 | NOTCH1 | 1 | TP53:VEGFA | 1 | HES4:MMP23B:RET |
| 8 | PEG3 | 1 | TNK2:TP53 | 1 | HES4:MMP23B:NOTCH2 |
| 8 | RET | 1 | TGFBR3:TNK2 | 1 | HES4:IGF1R:MMP23B |
| 8 | RPTOR | 1 | STAT1:TP53 | 1 | HDAC2:STAT1:TP53 |
| 8 | SPTBN1 | 1 | SPRY2:TP53 | 1 | HDAC1:NOTCH2:TP53 |
| 8 | TNK2 | 1 | RPTOR:TP53 | 1 | FLT1:MAP2K3:TP53 |
| 7 | BRCA1 | 1 | RET:TNKS | 1 | EP300:MYC:TP53 |
| 7 | FLT1 | 1 | RBPJL:TNKS | 1 | EP300:MYC:PEG3 |
| 7 | HES4 | 1 | RB1:SPEN | 1 | EP300:MAML1:RBPJL |
| 7 | IGF1R | 1 | PSEN1:TP53 | 1 | EGFR:PEG3:TP53 |
| 7 | MAPK7 | 1 | PRKCA:RET | 1 | EGFR:KRAS:MYC |
| 7 | MET | 1 | PIK3CA:SMAD4 | 1 | DTX1:ROS1:SSRP1 |
| 7 | MMP23B | 1 | PDK1:TP53 |  |  |
| 7 | MTOR | 1 | PDGFRA:TP53 |  |  |
| 7 | PDGFRA | 1 | NCOR1:SMAD4 |  |  |
| 7 | PIK3CG | 1 | MYC:YES1 |  |  |
| 7 | RBPJL | 1 | MYC:TP53 |  |  |
| 7 | SSRP1 | 1 | MYC:SPRY2 |  |  |
| 7 | STAT2 | 1 | MYC:PTK2 |  |  |
|  |  |  |  |  |  |

A summary of the node count of defective genes for each SOM clade appears in **Appendix Table II**. This table has been sorted according to defective gene counts, where the largest number of SOM nodes (n=2-7) have significant Student’s scores comparing tumor cells with and without defective IGF1R. This result is followed in decreasing order by CDC25A, MYC, NOTCH1, ZHX2, PTK2, RPTOR, SPTBN1 and BRAF. Noteworthy is the occurrence of the largest number of defective genes in meta-clades 9 and 26.

**Appendix Table II:**

Meta-clade

| **1** | **2** | **7** | **9** | **10** | **12** | **13** | **14** | **15** | **16** | **17** | **18** | **19** | **20** | **21** | **22** | **24** | **25** | **26** | **27** | **28** | **SUM** | **GENE** |
| --- | --- | --- | --- | --- | --- | --- | --- | --- | --- | --- | --- | --- | --- | --- | --- | --- | --- | --- | --- | --- | --- | --- |
| **1** | **2** | **0** | **4** | **3** | **20** | **16** | **19** | **53** | **1** | **0** | **0** | **1** | **2** | **8** | **2** | **13** | **19** | **17** | **0** | **7** | **207** | **IGF1R** |
| **2** | **2** | **4** | **0** | **48** | **0** | **22** | **0** | **14** | **3** | **0** | **0** | **21** | **29** | **0** | **0** | **1** | **7** | **0** | **0** | **4** | **160** | **CDC25A** |
| **1** | **0** | **3** | **27** | **0** | **0** | **17** | **0** | **1** | **4** | **12** | **10** | **7** | **4** | **5** | **11** | **0** | **2** | **18** | **9** | **11** | **146** | **MYC** |
| **0** | **2** | **0** | **0** | **9** | **7** | **14** | **18** | **12** | **3** | **1** | **0** | **1** | **0** | **0** | **17** | **6** | **9** | **14** | **2** | **12** | **130** | **NOTCH1** |
| **1** | **1** | **7** | **49** | **1** | **0** | **1** | **0** | **0** | **2** | **6** | **0** | **6** | **5** | **0** | **0** | **0** | **1** | **7** | **4** | **2** | **100** | **ZHX2** |
| **1** | **2** | **14** | **13** | **1** | **1** | **10** | **0** | **0** | **1** | **9** | **3** | **2** | **4** | **0** | **7** | **4** | **0** | **8** | **7** | **2** | **91** | **PTK2** |
| **4** | **4** | **1** | **0** | **1** | **7** | **13** | **10** | **9** | **0** | **0** | **0** | **8** | **1** | **5** | **12** | **1** | **0** | **3** | **3** | **6** | **91** | **RPTOR** |
| **0** | **3** | **0** | **0** | **0** | **1** | **2** | **4** | **3** | **14** | **1** | **0** | **4** | **0** | **0** | **9** | **0** | **4** | **21** | **12** | **7** | **86** | **SPTBN1** |
| **3** | **0** | **11** | **0** | **2** | **0** | **0** | **0** | **0** | **2** | **16** | **7** | **32** | **3** | **0** | **0** | **0** | **0** | **0** | **0** | **2** | **81** | **BRAF** |
| **0** | **1** | **0** | **0** | **1** | **4** | **9** | **7** | **1** | **2** | **2** | **3** | **3** | **1** | **4** | **21** | **1** | **1** | **7** | **0** | **10** | **79** | **STAT2** |
| **0** | **2** | **0** | **5** | **0** | **0** | **4** | **0** | **2** | **1** | **0** | **0** | **0** | **0** | **4** | **22** | **6** | **0** | **16** | **2** | **10** | **74** | **AXL** |
| **9** | **2** | **6** | **1** | **3** | **2** | **8** | **1** | **7** | **1** | **1** | **7** | **5** | **3** | **0** | **1** | **1** | **1** | **2** | **0** | **5** | **67** | **MTOR** |
| **0** | **5** | **0** | **7** | **0** | **2** | **4** | **6** | **2** | **0** | **4** | **0** | **7** | **1** | **0** | **0** | **0** | **1** | **14** | **2** | **5** | **64** | **PIK3CG** |
| **0** | **0** | **0** | **14** | **0** | **2** | **0** | **0** | **1** | **10** | **1** | **4** | **0** | **0** | **0** | **8** | **0** | **0** | **18** | **0** | **4** | **63** | **TNKS** |
| **0** | **2** | **1** | **32** | **0** | **0** | **1** | **0** | **0** | **0** | **1** | **0** | **0** | **0** | **0** | **3** | **0** | **0** | **9** | **4** | **3** | **60** | **MECOM** |
| **1** | **7** | **0** | **17** | **1** | **0** | **12** | **0** | **0** | **1** | **3** | **6** | **0** | **0** | **0** | **0** | **0** | **0** | **5** | **0** | **1** | **55** | **EIF5A2** |
| **10** | **3** | **7** | **12** | **1** | **0** | **0** | **0** | **0** | **0** | **1** | **0** | **0** | **3** | **0** | **2** | **0** | **0** | **0** | **1** | **0** | **54** | **MMP1** |
| **0** | **0** | **0** | **1** | **0** | **7** | **0** | **10** | **3** | **0** | **0** | **0** | **2** | **1** | **24** | **0** | **0** | **0** | **0** | **0** | **0** | **53** | **CDKN2A** |
| **0** | **5** | **0** | **0** | **0** | **5** | **1** | **2** | **4** | **2** | **0** | **2** | **1** | **7** | **0** | **3** | **0** | **2** | **11** | **4** | **3** | **53** | **EGFR** |
| **1** | **0** | **9** | **6** | **1** | **1** | **10** | **0** | **0** | **1** | **11** | **5** | **1** | **0** | **0** | **1** | **0** | **0** | **3** | **0** | **1** | **52** | **CYP11B1** |
| **1** | **0** | **9** | **6** | **1** | **1** | **10** | **0** | **0** | **1** | **11** | **5** | **1** | **0** | **0** | **1** | **0** | **0** | **3** | **0** | **1** | **52** | **CYP11B2** |
| **1** | **3** | **2** | **1** | **0** | **6** | **3** | **0** | **5** | **0** | **0** | **0** | **1** | **1** | **0** | **8** | **9** | **0** | **7** | **0** | **3** | **50** | **ATM** |
| **0** | **2** | **0** | **0** | **0** | **0** | **5** | **1** | **1** | **0** | **0** | **0** | **13** | **6** | **0** | **1** | **0** | **1** | **3** | **4** | **3** | **49** | **TEK** |
| **9** | **2** | **0** | **1** | **6** | **2** | **0** | **10** | **2** | **0** | **0** | **2** | **1** | **3** | **0** | **0** | **0** | **0** | **5** | **1** | **2** | **48** | **ABL1** |
| **0** | **1** | **0** | **8** | **0** | **11** | **2** | **4** | **2** | **0** | **1** | **0** | **0** | **0** | **3** | **0** | **0** | **0** | **2** | **0** | **2** | **45** | **NRAS** |
| **7** | **6** | **0** | **1** | **0** | **4** | **10** | **2** | **6** | **0** | **0** | **1** | **3** | **0** | **0** | **0** | **0** | **0** | **2** | **0** | **0** | **42** | **SPEN** |
| **0** | **5** | **0** | **0** | **3** | **1** | **2** | **1** | **3** | **3** | **0** | **0** | **1** | **1** | **0** | **2** | **2** | **0** | **2** | **0** | **3** | **40** | **NCOR2** |
| **0** | **3** | **1** | **0** | **0** | **0** | **0** | **1** | **1** | **15** | **0** | **0** | **3** | **0** | **0** | **0** | **0** | **0** | **3** | **0** | **2** | **31** | **MST1R** |
| **4** | **1** | **0** | **0** | **0** | **15** | **0** | **0** | **1** | **2** | **0** | **0** | **0** | **3** | **1** | **0** | **0** | **0** | **1** | **0** | **0** | **30** | **MSH2** |
| **0** | **0** | **1** | **13** | **0** | **0** | **0** | **0** | **0** | **1** | **0** | **0** | **0** | **0** | **0** | **0** | **0** | **0** | **4** | **5** | **3** | **28** | **EP300** |
| **5** | **2** | **4** | **0** | **3** | **0** | **4** | **0** | **0** | **0** | **0** | **0** | **1** | **1** | **0** | **0** | **1** | **0** | **2** | **0** | **0** | **27** | **APC** |
| **1** | **2** | **0** | **0** | **0** | **0** | **0** | **0** | **0** | **1** | **0** | **1** | **1** | **0** | **0** | **2** | **0** | **0** | **7** | **6** | **2** | **24** | **CDH1** |
| **0** | **4** | **0** | **0** | **0** | **0** | **0** | **0** | **3** | **0** | **0** | **0** | **0** | **0** | **0** | **3** | **7** | **0** | **2** | **1** | **2** | **23** | **MLH1** |
| **4** | **0** | **2** | **1** | **0** | **0** | **0** | **0** | **0** | **1** | **1** | **0** | **0** | **0** | **0** | **0** | **0** | **0** | **4** | **6** | **2** | **23** | **PEG3** |
| **0** | **2** | **5** | **1** | **1** | **0** | **0** | **0** | **0** | **1** | **0** | **0** | **2** | **0** | **0** | **0** | **0** | **0** | **0** | **0** | **1** | **22** | **ACVR2A** |
| **0** | **0** | **0** | **0** | **0** | **0** | **0** | **0** | **0** | **0** | **0** | **0** | **1** | **0** | **2** | **11** | **0** | **0** | **0** | **6** | **2** | **22** | **KRAS** |
| **0** | **3** | **2** | **0** | **0** | **0** | **0** | **0** | **0** | **9** | **2** | **0** | **1** | **0** | **0** | **0** | **0** | **0** | **2** | **0** | **0** | **21** | **MMP9** |
| **1** | **12** | **1** | **1** | **0** | **0** | **2** | **0** | **0** | **0** | **0** | **0** | **0** | **0** | **0** | **0** | **0** | **0** | **0** | **0** | **3** | **20** | **PIK3CA** |
| **7** | **2** | **0** | **0** | **0** | **1** | **0** | **0** | **3** | **0** | **0** | **0** | **0** | **1** | **2** | **0** | **3** | **0** | **0** | **0** | **1** | **20** | **RB1** |
| **0** | **0** | **0** | **3** | **0** | **0** | **2** | **0** | **0** | **6** | **0** | **3** | **1** | **4** | **0** | **0** | **0** | **0** | **0** | **0** | **1** | **20** | **RBPJL** |
| **2** | **4** | **0** | **0** | **0** | **0** | **0** | **0** | **0** | **0** | **0** | **0** | **0** | **1** | **0** | **0** | **0** | **0** | **0** | **0** | **0** | **18** | **ATR** |
| **1** | **1** | **1** | **0** | **0** | **4** | **0** | **0** | **0** | **1** | **4** | **1** | **0** | **1** | **0** | **1** | **0** | **0** | **0** | **0** | **0** | **18** | **MAP2K3** |
| **0** | **2** | **2** | **0** | **0** | **0** | **0** | **0** | **0** | **8** | **1** | **0** | **0** | **0** | **0** | **0** | **0** | **0** | **0** | **0** | **0** | **17** | **ALK** |
| **3** | **5** | **0** | **0** | **0** | **0** | **1** | **0** | **0** | **0** | **0** | **0** | **0** | **8** | **0** | **0** | **0** | **0** | **0** | **0** | **0** | **17** | **JAK3** |
| **1** | **0** | **1** | **6** | **0** | **1** | **3** | **0** | **0** | **2** | **2** | **0** | **0** | **0** | **0** | **0** | **0** | **0** | **0** | **0** | **0** | **16** | **BCR** |
| **1** | **1** | **1** | **0** | **0** | **0** | **0** | **0** | **0** | **7** | **1** | **1** | **1** | **0** | **0** | **0** | **0** | **0** | **0** | **0** | **0** | **16** | **FLT1** |
| **11** | **1** | **1** | **0** | **0** | **0** | **0** | **0** | **0** | **0** | **0** | **0** | **0** | **1** | **0** | **0** | **0** | **0** | **0** | **0** | **0** | **16** | **MAPK7** |
| **0** | **4** | **2** | **1** | **0** | **1** | **2** | **0** | **0** | **1** | **0** | **0** | **0** | **0** | **0** | **0** | **1** | **0** | **0** | **0** | **1** | **16** | **TSC2** |
| **0** | **0** | **0** | **1** | **0** | **3** | **0** | **2** | **0** | **0** | **0** | **0** | **1** | **1** | **6** | **0** | **0** | **0** | **0** | **0** | **0** | **15** | **CDKN2B** |
| **0** | **1** | **0** | **0** | **0** | **0** | **2** | **1** | **0** | **0** | **1** | **4** | **1** | **0** | **0** | **1** | **0** | **0** | **0** | **0** | **2** | **15** | **ITGB3** |
| **3** | **0** | **0** | **0** | **0** | **0** | **0** | **0** | **0** | **0** | **0** | **0** | **0** | **0** | **0** | **0** | **0** | **0** | **0** | **0** | **0** | **14** | **NF1** |
| **4** | **2** | **0** | **0** | **1** | **1** | **0** | **0** | **0** | **0** | **3** | **0** | **0** | **0** | **2** | **0** | **1** | **0** | **0** | **0** | **0** | **14** | **NOTCH3** |
| **5** | **3** | **1** | **0** | **0** | **0** | **0** | **0** | **0** | **0** | **0** | **0** | **0** | **1** | **0** | **0** | **0** | **1** | **0** | **0** | **0** | **14** | **SOX9** |
| **0** | **4** | **0** | **8** | **0** | **0** | **0** | **0** | **0** | **0** | **0** | **0** | **0** | **0** | **0** | **0** | **0** | **0** | **0** | **0** | **0** | **12** | **HES1** |
| **0** | **1** | **2** | **0** | **0** | **0** | **0** | **0** | **0** | **0** | **1** | **0** | **0** | **2** | **0** | **2** | **0** | **0** | **1** | **0** | **0** | **12** | **KDR** |
| **0** | **5** | **0** | **0** | **0** | **4** | **0** | **0** | **0** | **0** | **0** | **0** | **0** | **1** | **0** | **0** | **1** | **0** | **0** | **0** | **1** | **12** | **NOTCH2** |
| **9** | **0** | **0** | **0** | **0** | **0** | **0** | **0** | **0** | **0** | **0** | **0** | **0** | **0** | **0** | **0** | **0** | **0** | **0** | **0** | **0** | **12** | **PIK3R1** |
| **1** | **1** | **0** | **3** | **0** | **1** | **0** | **0** | **0** | **0** | **0** | **0** | **0** | **0** | **2** | **0** | **0** | **0** | **0** | **0** | **0** | **12** | **RET** |
| **0** | **1** | **1** | **6** | **0** | **0** | **0** | **0** | **0** | **0** | **0** | **0** | **0** | **0** | **0** | **1** | **0** | **0** | **0** | **0** | **0** | **11** | **E2F7** |
| **0** | **3** | **0** | **0** | **0** | **4** | **0** | **0** | **0** | **0** | **0** | **0** | **1** | **0** | **0** | **0** | **0** | **0** | **0** | **0** | **1** | **11** | **ROS1** |
| **1** | **3** | **1** | **5** | **0** | **0** | **0** | **0** | **0** | **0** | **0** | **0** | **0** | **0** | **0** | **0** | **0** | **0** | **0** | **0** | **0** | **11** | **TNK2** |
| **3** | **2** | **0** | **0** | **0** | **1** | **0** | **0** | **0** | **0** | **0** | **0** | **4** | **0** | **0** | **0** | **0** | **0** | **0** | **0** | **0** | **10** | **BLM** |
| **0** | **2** | **1** | **5** | **0** | **0** | **0** | **0** | **0** | **0** | **1** | **1** | **0** | **0** | **0** | **0** | **0** | **0** | **0** | **0** | **0** | **10** | **BMP7** |
| **0** | **3** | **3** | **1** | **0** | **0** | **0** | **0** | **0** | **0** | **0** | **0** | **0** | **0** | **0** | **0** | **0** | **0** | **0** | **0** | **0** | **10** | **MMP2** |
| **0** | **1** | **1** | **0** | **0** | **0** | **0** | **0** | **0** | **0** | **0** | **0** | **0** | **0** | **0** | **0** | **3** | **0** | **1** | **2** | **0** | **10** | **NCOR1** |
| **0** | **5** | **0** | **0** | **0** | **1** | **0** | **0** | **3** | **0** | **0** | **0** | **0** | **0** | **0** | **0** | **1** | **0** | **0** | **0** | **0** | **10** | **PAK4** |
| **0** | **1** | **0** | **1** | **0** | **0** | **0** | **0** | **0** | **0** | **0** | **0** | **0** | **0** | **0** | **1** | **0** | **0** | **2** | **0** | **0** | **10** | **TGFBR3** |
| **4** | **3** | **1** | **0** | **0** | **0** | **0** | **0** | **0** | **0** | **0** | **0** | **0** | **0** | **0** | **0** | **0** | **0** | **0** | **0** | **0** | **9** | **JAK2** |
| **0** | **2** | **0** | **0** | **0** | **0** | **0** | **0** | **1** | **0** | **0** | **0** | **4** | **0** | **0** | **0** | **0** | **2** | **0** | **0** | **0** | **9** | **MET** |
| **0** | **1** | **0** | **0** | **0** | **1** | **1** | **1** | **0** | **0** | **0** | **0** | **0** | **0** | **0** | **1** | **0** | **0** | **0** | **0** | **2** | **9** | **SMAD4** |
| **123** | **155** | **109** | **261** | **87** | **122** | **193** | **100** | **143** | **107** | **98** | **66** | **143** | **100** | **68** | **154** | **62** | **51** | **226** | **81** | **135** |  |  |

**Appendix Table III:**

| SOM node | NSC | FDA Name | MOA |
| --- | --- | --- | --- |
| 3,28 | NSC134727~ | Mitomycin | A2 |
| 3,28 | NSC26980~ | Mitomycin | A2 |
| 3,28 | NSC755880~ | Mitomycin | A2 |
| 6,3 | NSC79037~ | Lomustine | A6\|AlkAg |
| 1,25 | NSC6396~ | Thiotepa | A7 |
| 17,3 | NSC266046~ | Oxaliplatin | A7 |
| 2,24 | NSC281612~ | BEN | A7 |
| 1,24 | NSC119875~ | Cisplatin | A7\|AlkAg |
| 1,25 | NSC25154~ | Pipobroman | A7\|AlkAg |
| 1,25 | NSC34462~ | Uracil_mustard | A7\|AlkAg |
| 1,25 | NSC8806~ | Melphalan | A7\|AlkAg |
| 1,25 | NSC9706~ | Triethylenemelamine | A7\|AlkAg |
| 3,24 | NSC3088~ | Chlorambucil | A7\|AlkAg |
| 3,25 | NSC757087~ | Nitrogen_mustard | A7\|AlkAg |
| 3,25 | NSC757098~ | Melphalan | A7\|AlkAg |
| 3,25 | NSC762~ | Nitrogen_mustard | A7\|AlkAg |
| 17,6 | NSC354258~ | 8-Chloro-adenosine | AM |
| 39,16 | NSC633782~ | Simvastatin | AM |
| 40,16 | NSC754771~ | Itraconazole | AM |
| 4,10 | NSC326231~ | Buthionine_sulphoximine | AM |
| 43,9 | NSC758706~ | Simvastatin | AM |
| 36,19 | NSC127716~ | Decitabine | AM\|DNMT |
| 1,25 | NSC32065~ | Hydroxyurea | AM\|Dr |
| 11,3 | NSC32982~ | Curcumin | Apo |
| 2,14 | NSC714597~ | Imexon | Apo |
| 3,11 | NSC706363~ | Arsenic_trioxide | Apo |
| 3,3 | NSC759274~ | Arsenic_trioxide | Apo |
| 16,20 | NSC751548~ | Salinomycin | Apo\|Ab\|CD133 |
| 37,8 | NSC683863~ | Irofulven | Apo\|AlkAg\|RDI |
| 10,2 | NSC744912~ | Obatoclax | Apo\|BCL2 |
| 37,14 | NSC756877~ | 1st_Intermediate_to_TDP_665759 | Apo\|MDM2 |
| 21,13 | NSC747599~ | Nilotinib | BCR-ABL\|PK:YK |
| 5,16 | NSC743414~ | Imatinib | BCR-ABL\|PK:YK |
| 20,12 | NSC773263~ | Bafetinib | BCR-ABL\|PK:YK,FYN,LYN |
| 43,7 | NSC759877~ | Dasatinib | BCR-ABL\|PK:YK,PDGFR,KIT |
| 43,8 | NSC732517~ | Dasatinib | BCR-ABL\|PK:YK,PDGFR,KIT |
| 1,23 | NSC694501~ | BN-2629 | Db |
| 15,1 | NSC366140~ | Pyrazoloacridine | Db |
| 15,1 | NSC627168~ | Pyrazoloacridine | Db |
| 29,5 | NSC3053~ | Actinomycin_D | Db |
| 4,28 | NSC125066~ | Bleomycin | Db |
| 4,28 | NSC758612~ | Bleomycin | Db |
| 41,12 | NSC686288~ | Aminoflavone | Db\|DDI |
| 41,12 | NSC710464~ | AFP464 | Db\|DDI |
| 29,15 | NSC24559~ | Mithramycin | Db\|Rs |
| 4,28 | NSC698037~ | Pemetrexed | Df\|AM\|GARTF\|DHFR |
| 18,5 | NSC754230~ | Pralatrexate | Df\|CTNNB1\|DHFR |
| 18,4 | NSC740~ | Methotrexate | Df\|DHFR |
| 17,6 | NSC758186~ | Azacitidine | DNMT |
| 6,27 | NSC663249~ | Triapine | Dr\|CC |
| 2,28 | NSC613327~ | Gemcitabine | Ds |
| 36,17 | NSC63984~ | Cordycepin | Ds |
| 4,27 | NSC287459~ | Cytarabine | Ds |
| 4,27 | NSC63878~ | Cytarabine | Ds |
| 5,11 | NSC374551~ | Fenretinide | Ds |
| 5,28 | NSC606869~ | Clofarabine | Ds |
| 5,28 | NSC759857~ | Clofarabine | Ds |
| 8,23 | NSC760419~ | Fenretinide | Ds |
| 5,28 | NSC105014~ | Cladribine | Ds\|ADA |
| 5,28 | NSC118218~ | Fludarabine | Ds\|AM\|Dr |
| 5,28 | NSC124463~ | Fludarabine | Ds\|AM\|Dr |
| 5,28 | NSC312887~ | Fludarabine | Ds\|AM\|Dr |
| 4,27 | NSC639186~ | Raltitrexed | Ds\|DHFR\|TYMS |
| 18,7 | NSC752~ | 6-Mercaptopurine | Ds\|IMPDH2\|PPAT |
| 18,7 | NSC755~ | 6-Mercaptopurine | Ds\|IMPDH2\|PPAT |
| 18,7 | NSC757348~ | 6-Mercaptopurine | Ds\|IMPDH2\|PPAT |
| 18,7 | NSC759614~ | 6-Mercaptopurine | Ds\|IMPDH2\|PPAT |
| 16,4 | NSC19893~ | Fluorouracil | Ds\|Rs\|TYMS |
| 16,4 | NSC757036~ | Fluorouracil | Ds\|Rs\|TYMS |
| 3,28 | NSC758230~ | Floxuridine | Ds\|TYMS |
| 4,28 | NSC27640~ | Floxuridine | Ds\|TYMS |
| 18,4 | NSC767745~ | Pelitrexol | GARTF |
| 18,11 | NSC759852~ | Vorinostat | HDAC |
| 20,1 | NSC758774~ | Belinostat | HDAC |
| 34,21 | NSC701852~ | Vorinostat | HDAC |
| 37,19 | NSC630176~ | Depsipeptide | HDAC |
| 38,9 | NSC668814~ | kahalide_f | HDAC |
| 5,19 | NSC726630~ | Belinostat | HDAC |
| 34,12 | NSC732011~ | SR16157 | Ho |
| 36,28 | NSC34521~ | Dexamethasone_Decadron | Ho |
| 7,16 | NSC12198~ | Dromostanolone_Propionate | Ho |
| 43,4 | NSC749226~ | Abiraterone | Ho\|CYP17A1 |
| 7,20 | NSC71423~ | Megestrol_acetate | Ho\|PGR |
| 35,16 | NSC719276~ | Fulvestrant | Ho\|SERM |
| 9,8 | NSC747974~ | Raloxifene | Ho\|SERM |
| 12,9 | NSC749712~ | AT-13387 | HSP90 |
| 17,4 | NSC761390~ | By-Product_of_CUDC-305 | HSP90 |
| 30,5 | NSC255109~ | geldanamycin_analog | HSP90 |
| 30,5 | NSC707545~ | Alvespimycin | HSP90 |
| 41,12 | NSC728165~ | Lificguat | HYP\|HIF1\|EPAS1 |
| 9,27 | NSC763832~ | LOR-253 | KLF4\|MTF1 |
| 4,9 | NSC62343~ | 3-Bromopyruvate_(acid | Mito\|HK2 |
| 7,8 | NSC734325~ | Dimethylaminoparthenolide | NFkB |
| 28,8 | NSC757363~ | Tyrothricin | NonCan:Ant |
| 29,12 | NSC755892~ | Hydrastinine_HCl | NonCan:Antihemorrhagic |
| 6,25 | NSC756738~ | Digoxin | NonCan:CardG |
| 37,17 | NSC117186~ | Acetalax | NonCan:Cath |
| 37,17 | NSC59687~ | Acetalax | NonCan:Cath |
| 37,17 | NSC614826~ | bisacodyl | NonCan:Cath |
| 17,13 | NSC170984~ | Pimozide | NonCan:Psy |
| 27,4 | NSC759178~ | Pipamperone | NonCan:Psy |
| 36,28 | NSC92339~ | Fluphenazine | NonCan:Psy |
| 17,22 | NSC747856~ | Olaparib | PARP\|PARP1 |
| 40,14 | NSC753686~ | Olaparib | PARP\|PARP1 |
| 38,3 | NSC677083~ | okadaic_acid | Pase |
| 13,11 | NSC777193~ | LDK-378 | PK:ALK |
| 21,13 | NSC764134~ | Dabrafenib | PK:BRAF |
| 32,24 | NSC779217~ | AZD-9291 | PK:EGFR |
| 21,13 | NSC768068~ | Cobimetinib_(isomer_1 | PK:MAP2K,MAP2K1 |
| 41,23 | NSC697286~ | LY-294002 | PK:PIK3 |
| 43,21 | NSC618487~ | Staurosporine | PK:PRKCA |
| 37,19 | NSC656576~ | Midostaurin | PK:PRKCA,STK |
| 3,25 | NSC697726~ | RH1 | PK:ROS1 |
| 36,13 | NSC174939~ | Elesclomol | PK:ROS1 |
| 16,8 | NSC710297~ | PX-316 | PK:STK |
| 17,7 | NSC280594~ | Triciribine_phosphate | PK:STK |
| 18,25 | NSC638850~ | 7-Hydroxystaurosporine | PK:STK |
| 36,18 | NSC638646~ | 7-Hydroxystaurosporine | PK:STK |
| 34,18 | NSC701554~ | Seliciclib | PK:STK,CDK,CDK2,CDK7,CDK9 |
| 15,3 | NSC758247~ | Palbociclib | PK:STK,CDK,CDK4,CDK6 |
| 21,13 | NSC679828~ | PD-98059 | PK:STK,MAP2K,MAP2K1,MAP2K2 |
| 21,13 | NSC758246~ | Trametinib | PK:STK,MAP2K,MAP2K1,MAP2K2 |
| 17,23 | NSC606698~ | Rapamycin | PK:STK,MTOR |
| 37,16 | NSC733504~ | Everolimus | PK:STK,MTOR |
| 37,18 | NSC226080~ | Rapamycin | PK:STK,MTOR |
| 37,18 | NSC683864~ | Temsirolimus | PK:STK,MTOR |
| 21,13 | NSC741078~ | Selumetinib | PK:STK,YK,MAP2K,MAP2K1,MAP2K2 |
| 20,12 | NSC354462~ | Hypothemycin | PK:YK |
| 39,13 | NSC761191~ | AP-26113 | PK:YK,ALK,EGFR |
| 20,12 | NSC761431~ | Vemurafenib | PK:YK,BRAF |
| 43,8 | NSC761910~ | Ibrutinib | PK:YK,BTK |
| 25,28 | NSC715055~ | Gefitinib | PK:YK,EGFR |
| 25,28 | NSC745750~ | Lapatinib | PK:YK,EGFR,ERBB2 |
| 18,26 | NSC763371~ | Ruxolitinib | PK:YK,JAK,JAK1,JAK2 |
| 22,2 | NSC756645~ | Crizotinib | PK:YK,MET,ALK |
| 42,9 | NSC757436~ | Cabozantinib | PK:YK,MET,AXL,KIT,VEGFR,FLT3,RET |
| 43,20 | NSC761068~ | Cabozantinib | PK:YK,MET,AXL,KIT,VEGFR,FLT3,RET |
| 18,22 | NSC737754~ | Pazopanib | PK:YK,PDGFR,FGFR,KIT,VEGFR |
| 43,8 | NSC752782~ | Pazopanib | PK:YK,PDGFR,FGFR,KIT,VEGFR |
| 43,5 | NSC755980~ | Lenvatinib | PK:YK,PDGFR,FGFR,VEGFR,RET |
| 20,9 | NSC750690~ | Sunitinib | PK:YK,PDGFR,KIT,VEGFR |
| 22,15 | NSC758254~ | Ixazomib_citrate | PSM |
| 27,5 | NSC758252~ | Carfilzomib | PSM |
| 44,6 | NSC761385~ | Sonidegib | SMO\|Hg-Smo |
| 1,25 | NSC724998~ | LMP-400 | T1 |
| 2,28 | NSC609699~ | Topotecan | T1 |
| 2,28 | NSC616348~ | Irinotecan | T1 |
| 2,28 | NSC673596~ | 7-Ethyl-10-hydroxycamptothecin | T1 |
| 2,28 | NSC710270~ | Karenitecin | T1 |
| 2,28 | NSC725776~ | LMP776 | T1 |
| 2,28 | NSC728073~ | Irinotecan | T1 |
| 2,28 | NSC759263~ | Topotecan | T1 |
| 2,28 | NSC759878~ | Irinotecan | T1 |
| 6,27 | NSC708298~ | 7-Tert-butyl-10-hydroxycamptothecin | T1 |
| 7,1 | NSC629749~ | Lapachone | T1 |
| 1,26 | NSC122819~ | Teniposide | T2 |
| 1,26 | NSC141540~ | Etoposide | T2 |
| 1,26 | NSC246131~ | Valrubicin | T2 |
| 1,26 | NSC757804~ | Etoposide | T2 |
| 1,26 | NSC758255~ | Teniposide | T2 |
| 1,27 | NSC123127~ | Doxorubicin | T2 |
| 1,27 | NSC256942~ | Epirubicin | T2 |
| 1,27 | NSC756717~ | Daunorubicin | T2 |
| 1,27 | NSC758667~ | Teniposide | T2 |
| 1,27 | NSC759155~ | Doxorubicin | T2 |
| 1,27 | NSC759195~ | Epirubicin | T2 |
| 1,27 | NSC82151~ | Daunorubicin | T2 |
| 1,28 | NSC279836~ | Mitoxantrone | T2 |
| 1,28 | NSC301739~ | Mitoxantrone | T2 |
| 1,28 | NSC83142~ | Daunorubicin | T2 |
| 2,27 | NSC256439~ | Idarubicin | T2 |
| 27,6 | NSC264137~ | Elliptinium_Acetate | T2 |
| 4,26 | NSC169780~ | Dexrazoxane | T2\|NonCan:Cardioprotective |
| 2,22 | NSC320846~ | Batracylin | T2\|T1 |
| 27,4 | NSC707389~ | Eribulin_mesilate | Tu |
| 28,3 | NSC109874~ | Benzimate | Tu |
| 31,3 | NSC376128~ | Dolastatin_10 | Tu |
| 27,5 | NSC759174~ | Vincristine | Tu\|Tu-frag |
| 28,4 | NSC760087~ | Vinorelbine | Tu\|Tu-frag |
| 28,6 | NSC608210~ | Vinorelbine | Tu\|Tu-frag |
| 28,6 | NSC757384~ | Vinblastine | Tu\|Tu-frag |
| 30,3 | NSC49842~ | Vinblastine | Tu\|Tu-frag |
| 30,6 | NSC90636~ | Vinblastine | Tu\|Tu-frag |
| 32,1 | NSC67574~ | Vincristine | Tu\|Tu-frag |
| 28,3 | NSC628503~ | Docetaxel | Tu\|Tu-stab |
| 28,3 | NSC759850~ | Docetaxel | Tu\|Tu-stab |
| 28,4 | NSC125973~ | Paclitaxel | Tu\|Tu-stab |
| 28,4 | NSC758645~ | Paclitaxel | Tu\|Tu-stab |
| 3,28 | NSC75520~ | Tfdu | TYMS |
| 4,28 | NSC697912~ | 5-fluoro_deoxy_uridine_10mer | TYMS |

**Appendix Figures 3 and 4** display additional examples for defective genes PIK3RI and IGF1R, respectively.


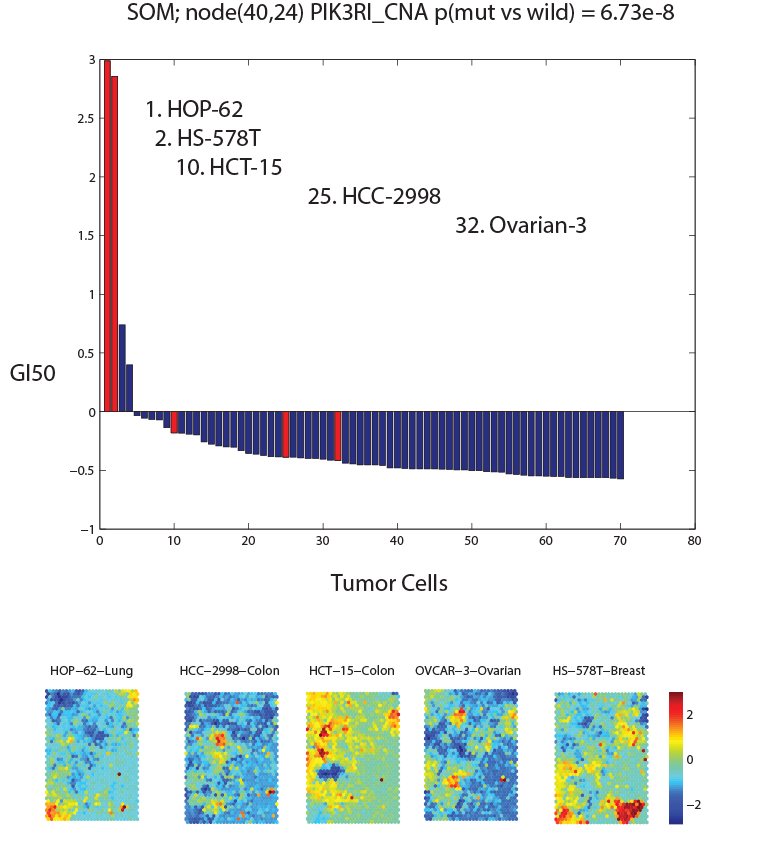


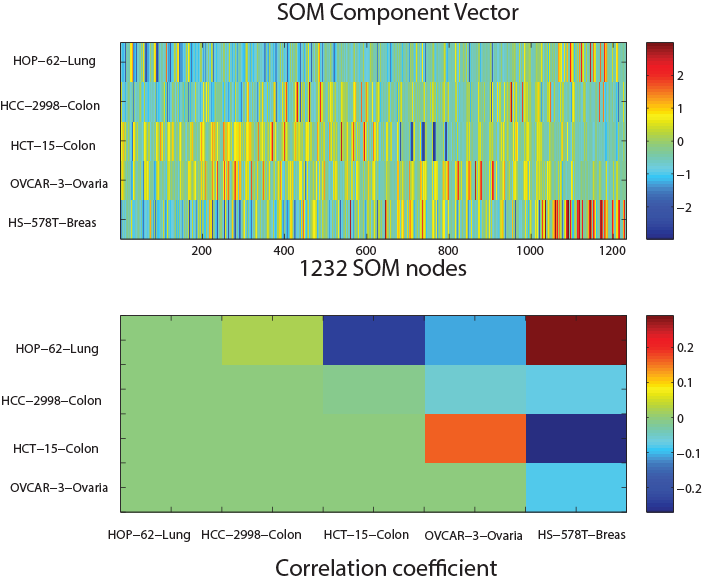


**Appendix Figure 3**: Upper panel displays GI50_codebook_ for SOM_40,24_, ordered from most to least chemosensitivity. The 5 tumor cells with defective PIK3R1 appear as red bars. Second panel displays SOM_component_ for the 5 tumor cells with defective PIK3R1. SOM nodes are colored spectrally from highest chemosensitivity (red) to lowest chemosensitivity (blue). Third panel displays, in linear form, SOM_components_. Lower panel displays the pairwise Pearson correlation coefficients for the 5 SOM_components_. HOP-62_HS-578T; r_15_=0.29, p_15_=1.50e-25, HCT-15_OVCAR-3; r_34_=0.17; p_34_=6.1e-9


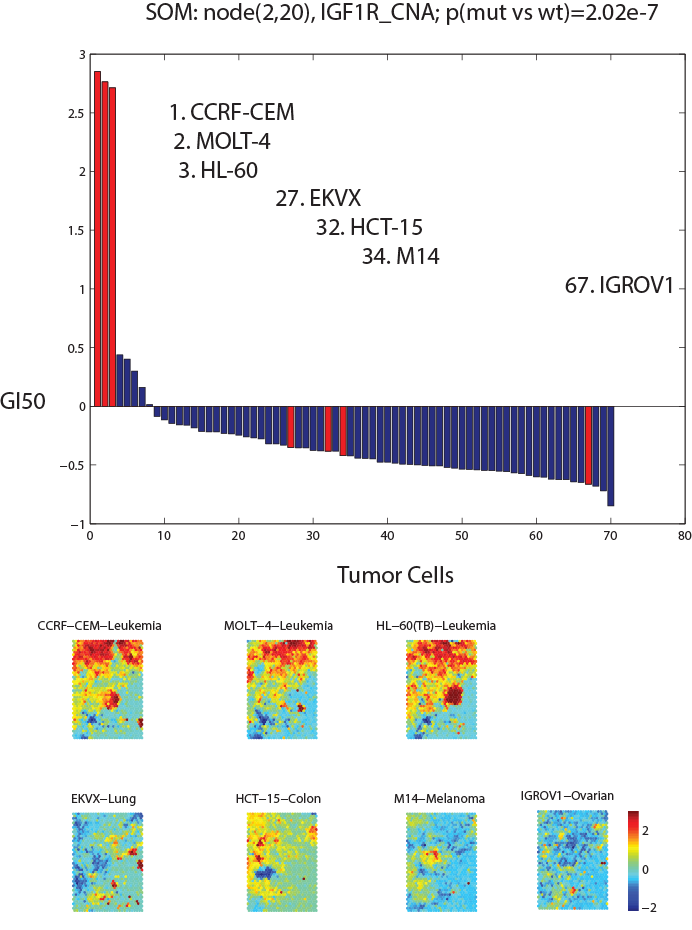


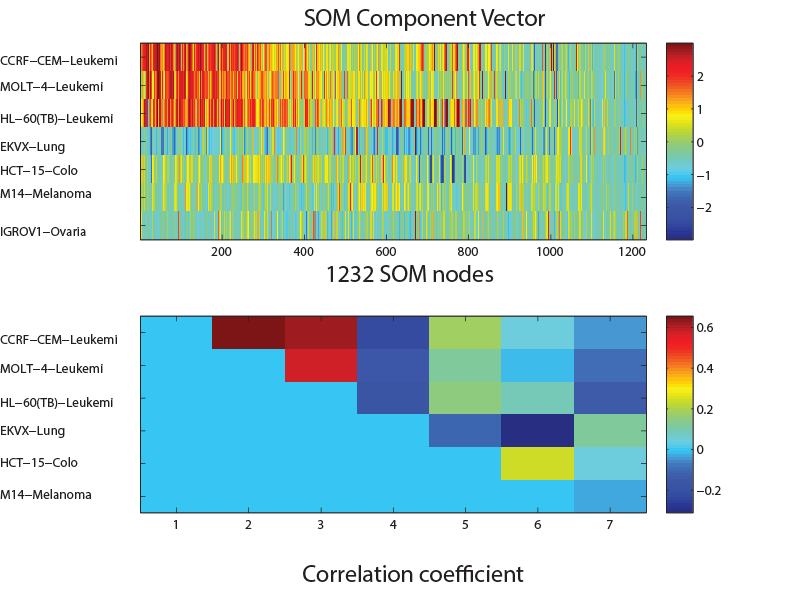


**Appendix Figure 4**: Upper panel displays GI50_codebook_ for SOM_2,20_, ordered from most to least chemosensitivity. The 7 tumor cells with defective IGFR1 appear as red bars. Second panel displays SOM_component_ for the 7 tumor cells with defective IGFR1. SOM nodes are colored spectrally from highest chemosensitivity (red) to lowest chemosensitivity (blue). Third panel displays the linear form of SOM_component_. Lower panel displays the pairwise Pearson correlation coefficients for the SOM_components_. r12=0.66 ; p12=8.83e-152 r13= 0.62 p13=3.4e-129 r15=0.19 p15=8.6e-11 r23=0.56 p23=7.8e-105 r56=0.25 p56=2.32e-18. This result supports an association between defective IGFR1 and enhanced chemosensitivity across the upper SOM region.

**Appendix Table IV: SOM meta-clades 1,2,4 and 7**

**Appendix Table V. SOM meta-clades 19 and 28**

**Appendix Table VI. SOM meta-clades 8 and 14**

**Appendix Table VII. SOM meta-clades 16,17 and 18**

**Appendix Table VIII. SOM meta-clade 9**

**Appendix Table IX: SOM meta-clades 11 and 12**

**Appendix Table X. SOM meta clades 23,24 and 25**

**Appendix Figure 5.** CBioPortal Display for defective genes in the NCI60. See Legend at the bottom for type of defective gene.


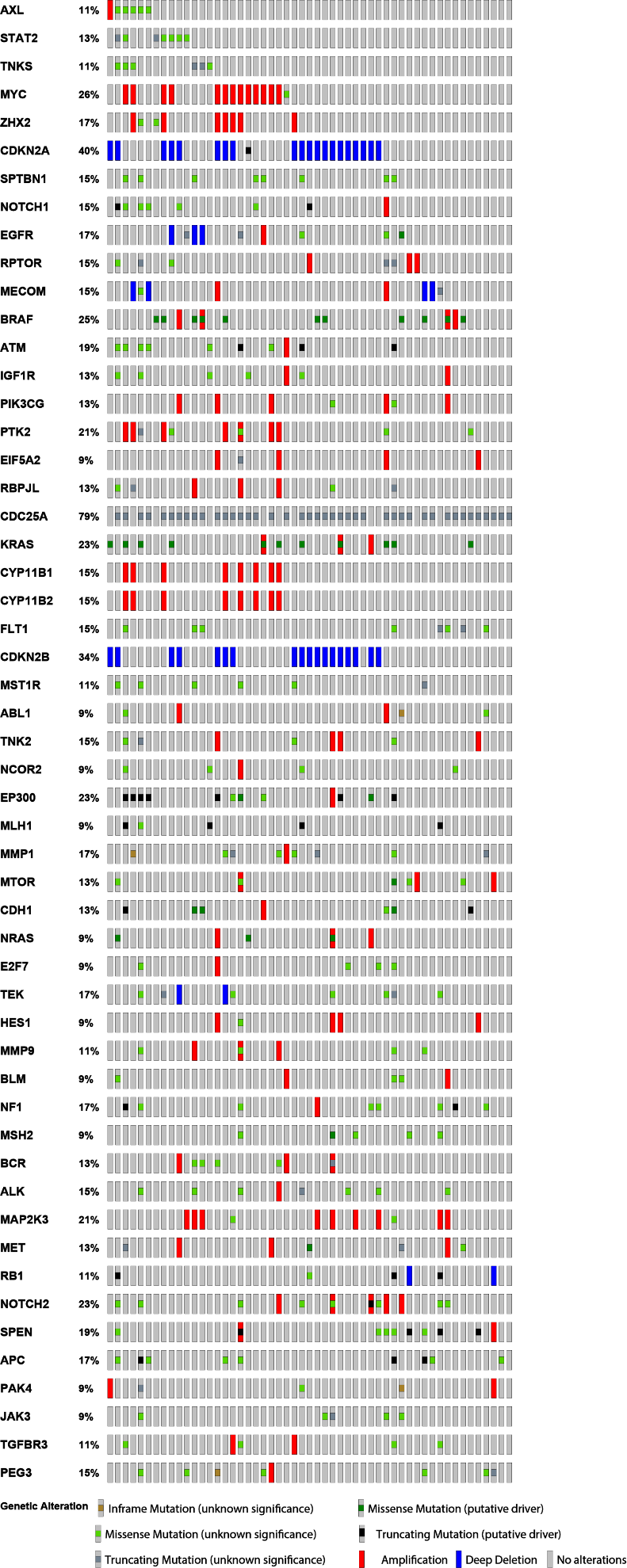

Supplement: S2 File — (DOCX) [file pone.0243336.s006.docx]
