# Supplementary material for: Bioinformatic analysis linking genomic defects to chemosensitivity and mechanism of action
Source: PLoS One. 2021 Apr 28;16(4):e0243336. doi: 10.1371/journal.pone.0243336 (PMC8081165; doi:10.1371/journal.pone.0243336)
Supplement: Attachment — Submitted filename: review.docx [file pone.0243336.s007.docx]

**OVERALL**

This manuscript combines three data sources: drug response data on the NCI60 cell lines which is clustered using self-organizing maps, gene alteration data on the NCI60 cell lines, and annotations of the mechanism of action (MOA). The primary goal is to relate disrupted or defective genes to mechanisms of action using the clustered drug compounds as an intermediary. The methods do not appear to be particularly novel (various clustering techniques, t-tests, and Fisher's Exact Tests) and many of the identified genes are already well-known as drivers of cancer and as targets of specific therapies. Nevertheless, there is utility in terms of the scope of the analysis. Unfortunately, the methods are not described in adequate detail, and there appears to have been no adjustment to account for multiple testing. Thus, in its present form, it is not possible to fully tell if the results are actually correct. Moreover, the results section appears to contain a substantial amount of discussion. Finally, the results-discussion appear overly long, giving the impression of a "data dump" without adequate attention having been paid to highlight the most significant aspects of the findings.

**MAJOR COMMENTS**

[1] A fundamental problem with this manuscript is that it would be completely impossible for anyone to replicate the results. The author never explains what the DTP is. This reviewer happens to know that it is the Department of Therapeutic Programs at the NIH. Going to their web site and guessing that (1) I need to follow the “bulk downloads” link and (2) I need to follow the "NCI60 Growth Inhibition Data" link gets me to a page with another decision. Which release of this data was used? October 2020? June 2020? Something else? Downloading the October 2020 release, one learns a couple of things. First, it contains data on about 55K compounds, more than the 53K mentioned in the manuscript. Second, it contains data on 159 cell lines, not just the 59 that are part of the NCI60. Leaving the reader in the dark about exactly which data were used (even if it is publicly available) is unacceptable.

[2] In general, the methods are also described too vaguely and with too little detail to allow a reader to replicate the results.

[3] Was the CV computation used for filtering compounds based on the negative log-transformed GI50 data, or was it computed on some other scale?

[4] Please describe in more detail the heuristic that was used to decide on the size of the SOM grid, or provide a complete reference.

[5] The text twice mentions a manuscript by "Holbeck et al." The first time, it does not link to a reference in the bibliography. The second time, it links to reference #19, by Whyte and Holbeck. Please fix this.

[6] I don't understand the methods (described on page 10 of the document for review, which contains lots of introductory pages before the manuscript actually starts) associated with Box D. Since the nodes in the SOM map contain drugs, how are we able to label some of those nodes based on the mutations of tumor cell lines? I _think_ the point is that the "codebook" for each node consists of an average (over the compounds assigned to the node) expression level for each cell line, which is followed by a t-test comparing the expression between cell lines with and without an abnormality. This step seems to be followed by a permutation test to compute empirical p-values. But this means that they have performed more than 4.5 million statistical tests, without making any kind of adjustment for multiple testing. Why should I believe that a cutoff of "p < 0.05" produces anything but random noise in this context? In fact, he may have found *fewer* significant nodes than expected by chance (when you consider that you are performing 368 tests, each with a 5% chance of being picked randomly, per node).

[7] Why was the association between clusters and defective genes performed at the level of 1232 SOM nodes (clades) and not at the level of 28 meta-clades or 7 meta-clade groupings?

[8] The caption for Figure 1 needs to explain/define the numerous abbreviations and acronyms employed. One also suspects that the duplication of "T1" in Box B should actually read "T1, T2".

[9] Figure 2 should include a colorbar to make it easier for readers to interpret the SOM plot. It would help of the author prevented the plot title from overlapping the list of compounds. It would also help to label the subpanels (with something like A, B, C) and to make it clearer why there are two histograms and how they differ. This is especially true since the figure caption does not discuss the histograms. Also, both histograms should use "inter-node" and not the spurious plural.

[10] None of the graphical methods in Figure 3 appear to contain a compelling reason to select the numbers of clusters ("meta-clades") proposed by the author. Nor do they justify the collapse of those categories into seven "meta-clade groupings".

[11] Using a gradient-based color scheme to label discrete meta-clades and meta-clade groupings in Figure 4 is potentially highly misleading. For one thing, it is extremely difficult to tell if the color boundaries actually align with valid branches in the accompanying dendrogram, especially for the gray-scale meta-clade groupings. For visualization, the author might want to consider the Polychrome R package, which can produce a wider variety of distinct colors for displaying discrete classes.

[12] More importantly, he might want to consider other clustering methods (K-means? PAM?) in light of the fact that the hierarchically defined meta-clades frequently break up into distinct subregions of the SOM display, indicating an incompatibility of clusters between the two methods. As sort-of noted by the author, this disparity is probably caused by changing distance metrics. Both hierarchical clustering and PAM can use the exact same distance metric that was applied when performing SOM clustering. And it would be important to use the same distance metric when applying methods to estimate the number of clusters. Visualizing the data with alternative methods such as t-SNE or UMAP might also provide additional insight into whether one should believe the clustering results.

[13] it would be useful to see a version of the original SOM plot (from Figure 2) with the meta-clade boundaries (from Figure 4) superimposed. After all, the original plot indicates the relative distance between neighboring SOM nodes, so it would provide greater support for the clustering if the boundaries tended to run through the yellow areas where neighbors aren't particularly close.

[14] From the figure and its caption, it is not clear what we are supposed to learn from Figure 5. My immediate reaction is that ABL1 is not particularly associated with GI50 values in this SOM node. What I get from this plot is that perhaps one should be using a Wilcoxon rank sum test instead of a t-test to associate defective genes with SOM nodes. I have a similar reaction to Figure 6.

[15] Figure 7 appears to be somewhat misleading by over-representing the amount of data associated with the CellMiner MOA annotations. While the SOM clusters were created from almost 47000 compounds, the MOA data is only available for 104 compounds, which is only about 0.22% of the total compounds considered. Each of those 104 compounds is then apparently used up to eight times. Is there any statistical significance to any of these "interpretive" assignments?

[16] Frankly, I am unclear on exactly how many times Fisher's Exact test was used to associate MOA's with defective genes. Among other things, I have completely lost track of whether these counts are based on SOM nodes, meta-clades, or meta-clade groups. And, once again, there is no sign that any corrections to p-values have been made to account for multiple testing.

[17] Beginning on page 17 of the document for reviewers, the author drifts from presenting results to discussing their interpretation. It would be easier for the reader if these parts of the manuscript remained more clearly separated.

[18] It would be nice if the font sizes in the numerous tables were large enough to be readable.
