# Supplementary material for: Bioinformatic analysis linking genomic defects to chemosensitivity and mechanism of action
Source: PLoS One. 2021 Apr 28;16(4):e0243336. doi: 10.1371/journal.pone.0243336 (PMC8081165; doi:10.1371/journal.pone.0243336)
Supplement: Attachment — Submitted filename: PONE_reviewer_comments_revised.docx [file pone.0243336.s008.docx]

*PONE-D-20-35751*

*Bioinformatic Analysis Linking Genomic Defects to Chemosensitivity and Mechanism of Action*

*PLOS ONE*

*The reviewers are of the opinion that the methods section is not described enough for the study to be replicated. A detailed introduction and methods section is warranted. Further, all the changes in figures as well as in the results and discussion sections as suggested by the reviewers need to be included. The main findings of the study has to be highlighted clearly.*

I thank you and the Reviewers for their many helpful comments. Guided by these comments, the manuscript has undergone a major reorganization, designed to clarify the METHODS and identify the major RESULTS of this study. Towards these goals, the tracked version has been included in the revised material, however the extent of editing makes interpretation difficult. Specific comments will be provided in the following responses. Please note that some of the material has been duplicated in the responses to each Reviewer.

I would like to emphasize my thanks to the reviewers. Their comments have resulted in a substantially improved manuscript. I look forward to your response.

*Comments from the Reviewer 1 (in case the author can't access the attached file):*

*OVERALL*

*This manuscript combines three data sources: drug response data on the NCI60 cell lines which is clustered using self-organizing maps, gene alteration data on the NCI60 cell lines, and annotations of the mechanism of action (MOA). The primary goal is to relate disrupted or defective genes to mechanisms of action using the clustered drug compounds as an intermediary. The methods do not appear to be particularly novel (various clustering techniques, t-tests, and Fisher's Exact Tests) and many of the identified genes are already well-known as drivers of cancer and as targets of specific therapies. Nevertheless, there is utility in terms of the scope of the analysis. Unfortunately, the methods are not described in adequate detail, and there appears to have been no adjustment to account for multiple testing. Thus, in its present form, it is not possible to fully tell if the results are actually correct. Moreover, the results section appears to contain a substantial amount of discussion.*

Please see the comments below.

*Finally, the results-discussion appear overly long, giving the impression of a "data dump" without adequate attention having been paid to highlight the most significant aspects of the findings.*

The revised manuscript represents a major reorganization of material and descriptions (DATA, METHODS, RESULTS). The results provided for meta-clade groups A-G are intended to summarize the findings (Panels A-D in the figure reported for each group) and provide literature support for their importance. To provide adequate literature support the referenced material represents only a subset of available information. Hopefully the impression of a “data dump” has been removed.

*MAJOR COMMENTS*

*[1] A fundamental problem with this manuscript is that it would be completely impossible for anyone to replicate the results. The author never explains what the DTP is. This reviewer happens to know that it is the Department of Therapeutic Programs at the NIH. Going to their web site and guessing that (1) I need to follow the “bulk downloads” link and (2) I need to follow the "NCI60 Growth Inhibition Data" link gets me to a page with another decision. Which release of this data was used? October 2020? June 2020? Something else? Downloading the October 2020 release, one learns a couple of things. First, it contains data on about 55K compounds, more than the 53K mentioned in the manuscript. Second, it contains data on 159 cell lines, not just the 59 that are part of the NCI60. Leaving the reader in the dark about exactly which data were used (even if it is publicly available) is unacceptable.*

I apologize for the confusion and have included the following to the revised manuscript:

*[2] In general, the methods are also described too vaguely and with too little detail to allow a reader to replicate the results.*

I agree. The methods section has been reorganized to separately treat the three datasets (chemosensitivity, defective genes and MOA). Withing these sections the methods of analysis are provided in detail with examples as illustrations. These sections comprise **METHODS: data clustering**, **METHODS: identification of SOM_DTP_ nodes with enhanced chemosensitivity** and **METHODS: mapping MOA to SOM_DTP_**. I appreciate each Reviewer’s concerns and believe the revised version provides adequate details of both the data sources and methods of analysis.

*[3] Was the CV computation used for filtering compounds based on the negative log-transformed GI50 data, or was it computed on some other scale?*

Data filtering was based on the negative log-transformed GI50 data (see the comment above).

*[4] Please describe in more detail the heuristic that was used to decide on the size of the SOM grid, or provide a complete reference.*

The SOM tools for use within MATLAB provides a set of utilities for SOM-related calculations (Self-Organizing Map in Matlab: The SOM toolbox, Vesanto et al. [1]. These tools are derived from the SomPak download (Essentials of the self-organizing Maps, Kohonen [2]. Examination of the MATLAB programs for SOM initialization (specifically som_init.m) finds a heuristic that uses the ratio of the first and second principal components to determine map dimensions. The y-axis dimension is calculated as round(sqrt(munits/ratio*sqrt(0.75))), where munits=5*nsamples^0.543. The x-axis dimension is calculated as round(munits/y-dimension). This heuristic appears to be derived from the developers of SomPak, based on their usage. The sqrt(0.75) multiplier is explained as follows, “in the hexagonal lattice, the side lengths are not directly proportional to munits ( = 5*nsamples^0.543) since the units on the y-axis are squeezed together by a factor of sqrt(0.75)”. SOM dimensions of 44x28 were determined for the GI50 dataset used in this analysis.

A condensed version of this description now appears as a footnote:

The ratio of the first and second principal components of the data is used to determine map dimensions. The y-axis dimension is calculated as round(sqrt(munits/ratio*sqrt(0.75))), where munits=5*nsamples^0.543. The x-axis dimension is calculated as round(munits/y-dimension). This heuristic appears to be derived from the developers of SomPak, based on their usage. The sqrt(0.75) multiplier is explained as follows, “in the hexagonal lattice, the side lengths are not directly proportional to munits ( = 5*nsamples^0.543) since the units on the y-axis are squeezed together by a factor of sqrt(0.75)”.

*[5] The text twice mentions a manuscript by "Holbeck et al." The first time, it does not link to a reference in the bibliography. The second time, it links to reference #19, by Whyte and Holbeck. Please fix this.*

Thanks. Fixed.

*[6] I don't understand the methods (described on page 10 of the document for review, which contains lots of introductory pages before the manuscript actually starts) associated with Box D. Since the nodes in the SOM map contain drugs, how are we able to label some of those nodes based on the mutations of tumor cell lines? I _think_* *the point is that the "codebook" for each node consists of an average (over the compounds assigned to the node) expression level for each cell line,* *which is followed by a t-test comparing the expression between cell lines with and without an abnormality. This step seems to be followed by a permutation test to compute empirical p-values**. But this means that they have performed more than 4.5 million statistical tests, without making any kind of adjustment for multiple testing. Why should I believe that a cutoff of "p < 0.05" produces anything but random noise in this context? In fact, he may have found *fewer* significant nodes than expected by chance (when you consider that you are performing 368 tests, each with a 5% chance of being picked randomly, per node).*

The reviewer is correct. Additional details added to the **METHODS** should improve this portion of the manuscript.

**METHODS: identification of SOM_DTP_ nodes with enhanced chemosensitivity**

SOM_DTP_ nodes are analyzed for enhanced chemosensitivity of tumor cells with versus without defective genes. Each GI50_codebook_ is divided into subsets comprising tumor cells with (GI50_defective_) and without (GI50_wild-type_) a defective gene. A Student’s t-test is used to identify cases of relatively higher chemosensitivity for GI50_defective_ versus GI50_wild-type_. SOM_DTP_ nodes with Student’s p-values less than or equal to 0.05 were further assessed for statistical significance by bootstrap resampling[3, 4]. Each node’s GI50_codebook_ was randomly shuffled and a Student’s t-test performed, while maintaining the tumor cell’s wild-type and defective gene status. One-thousand trials were conducted for each GI50_codebook_ and a p-value was estimated by counting the number of times the shuffled p-value was less than the initial, unshuffled, p-value. Dividing this value by 1000 yields an estimate for the probability of the observed p-value occurring by chance. SOM_DTP_ nodes with measured p-values less than 0.05 and below their estimated chance occurrence were accepted for further analysis. Sixty-five percent (65%, n=635) of the 1232 SOM nodes pass this criterion and account for 121 defective genes.

1. *the point is that the "codebook" for each node consists of an average (over the compounds assigned to the node) expression level for each cell line*

Each codebook vector represents the average GI50 values for the compounds assigned to a SOM_DTP_ node.

2. *which is followed by a t-test comparing the expression between cell lines with and without an abnormality.*

Correct, when ‘expression’ is replaced by ‘GI50 values’*.*

3. *This step seems to be followed by a permutation test to compute empirical p-values.*
4. *But this means that they have performed more than 4.5 million statistical tests, without making any kind of adjustment for multiple testing. Why should I believe that a cutoff of "p < 0.05" produces anything but random noise in this context? In fact, he may have found *fewer* significant nodes than expected by chance (when you consider that you are performing 368 tests, each with a 5% chance of being picked randomly, per node).*

The following has been added to the end of **METHODS: identification of SOM_DTP_ nodes with enhanced chemosensitivity**

The Students t-statistic represents the significance when comparing the chemosensitivity of a SOM_DTP_ node for tumor cells with, versus without, defective genes. Parametric tests, such as the Student’s t-test, are applicable over non-parametric tests (Wilcoxon/Whitney/Mann, Kruskal-Wallis) when the underlying sample distribution is known and normal. The data analyzed here represents a strongly normal distribution (p < 0.001, lognormal test) with small deviations at the tails from a linear log normal quantile-quantile plot; supporting the use of a parametric statistic.

The application of a bootstrap procedure to cases with a significant Student’s t-test is applied as a correction against Type I error for the following reasons. First, a bootstrap method can be used to estimate the sampling distribution of GI50_codebook_ for each SOM_DTP_ node. This test utilizes the node’s codebook vector as the initial sample representative and applies a bootstrap procedure to estimate the sampling distribution. Since 1000 samples were used, the p-value estimate corrects the empirical estimate using a division by 1000. This correction parallels multiple test corrections for traditional statistics[5]. Second, in this design there are 1000 statistical tests performed for each of the 1232 SOM_DTP_ nodes. An important caveat of jointly using GI50_codebook_ to create some type of correction for multiple test is their lack of independence [2, 6]. This non-independence is disallowed when applying a Bonferroni, Holm or Benjamini-Hochberg [7] corrections. None-the-less, the long-standing debate continues to exist about bootstrap applications, possible overestimation of ‘true’ values, and appropriate corrections for random noise[8].

*[7] Why was the association between clusters and defective genes performed at the level of 1232 SOM nodes (clades) and not at the level of 28 meta-clades or 7 meta-clade groupings?*

Each SOM_DTP_ node is assessed for differential chemosensitivity of each defective gene. The significant SOM_DTP_ nodes are catalogued and used for later analysis. The following example for *ABL1* and *KRAS*, added to the text, illustrates the process.

**Figure 4**: **Panels A** and **B** display significant chemosensitive SOM_DTP_ nodes (projected as their t-statistic from a Student’s t-test; blue:least, red:most significant) for tumor cells with defective *ABL1* and *KRAS*, respectively. **Panel C** displays the 28 SOM_DTP_ meta-clades.

*[8] The caption for Figure 1 needs to explain/define the numerous abbreviations and acronyms employed. One also suspects that the duplication of "T1" in Box B should actually read "T1, T2".*

Thanks for the comment. **ABBREVIATIONS** are now listed as a separate item in the manuscript. I would add that the original Figure 1, with the separate Boxes, has been eliminated in favor of separate headings for each data type and the associated analysis. Hopefully the confusion over this original Figure 1 will be eliminated.

*[9] Figure 2 should include a colorbar to make it easier for readers to interpret the SOM plot. It would help of the author prevented the plot title from overlapping the list of compounds. It would also help to label the subpanels (with something like A, B, C) and to make it clearer why there are two histograms and how they differ. This is especially true since the figure caption does not discuss the histograms. Also, both histograms should use "inter-node" and not the spurious plural.*

Thanks for the suggestion. This Figure has now been separated into Panels **A-D** and moved to **RESULTS: SOM_DTP_**.

**Figure 8: Panel A.** SOM_DTP_ is colored according to similarity of GI50_codebooks_, where the most similar node neighbors are displayed in deep red and the most dis-similar node neighbors appear in bright yellow (see vertical bar adjacent to SOM_DTP_). The 28 optimal meta-clade boundaries are displayed as a black line, with the boundaries of the 7 meta-clade groups super-imposed as a white line. FDA approved compounds are projected onto SOM_DTP_ as blue hexagons, where hexagons are sized according to the number of FDA agents appearing in any node. **Panel B** displays the between node GI50_codebook_ Euclidean distances for nodes with FDA compound projections (top) and without (bottom). **Panel C** lists FDA compound names grouped by 28 meta-clades. **Panel D** displays SOM_DTP_ with FDA compounds (blue hexagons), meta-clade boundaries (solid lines) and meta-clade labels as numbers. FDA approved projections to SOM_DTP_ nodes are listed in master_appendix sheet **appendix_Table_III**.

*[10] None of the graphical methods in Figure 3 appear to contain a compelling reason to select the numbers of clusters ("meta-clades") proposed by the author.* *Nor do they justify the collapse of those categories into seven "meta-clade groupings".*

The section **METHODS: data clustering** has been included with the following explanation.

While each SOM_DTP_ node represents a cluster of GI50_NCI60_ values, a more global, lower resolution representation, that optimally groups SOM_DTP_ nodes into meta-clades, is proposed. Three state-of-the art procedures are used to determine the optimal number of meta-clades; the **elbow** method minimizes the within-cluster sum of squares, WSS (a measure of within cluster similarity) and maximizes the between-cluster sum of squares (a measure of how separated each cluster is from the others), the **silhouette** method computes the average silhouette of observations for different numbers of clusters (selecting the optimal cluster size that maximizes the average silhouette) and the **gap_statistic** method [9] determines the total within intra-cluster variation for different numbers of clusters (selecting the cluster size that maximizes the gap_statistic). **Figure 1** displays the results for these three methods applied to GI50_codebooks_. The optimal cluster size is indicated by the vertical lines in each plot. The **elbow** and **gap** methods rely on an inflection point on each curve for optimal cluster size, while the **silhouette** method seeks the largest value for silhouette width. **Panel A** displays WSS as circles for the **elbow** method and the first derivative of WSS, normalized by the local average WSS, as triangles. The maximum value of the derivative of WSS occurs for 28 clades. The **silhouette** method yields a maximum value at 26 clusters (cf. **Panel B**). The criterion for optimal cluster size using the **gap** statistic seeks the smallest number of clusters such that the gap statistic is within one standard deviation of the next gap statistic: Gap(k)≥Gap(k + 1)−sd_(k+1)_ (displayed as triangles in **Panel C**), yields 28 clusters as optimal. **Panel D** displays the GI50_codebook_ cluster dendrogram using the cutree tool [10] to group the dendrogram into 28 meta-clades (red lines) and 7 meta-clades (green lines). Based on these results a value of 28 was selected for the optimal number of meta-clades used in this analysis. The rationale for cutting the dendrogram at 7 clusters will be provided later in the analysis of MOAs.

**Figure 1**. Results for selecting optimal cluster size using the **elbow** method (**Panel A**), **silhouette** method (**Panel B**) and the **gap**_**statistic** method (**Panel C**). Selection for the **elbow** method is based on the largest local derivative of the within groups sum of squares (**Panel A** triangles), the maximum silhouette width (**Panel B**) and the first non-negative value for Gap(k)-(Gap(k + 1)−sd_k+1_) (**Panel C** triangles). These results indicate an optimal number of clusters in the 26-28 range. **Panel D** displays the GI50_codebook_ dendrogram (Euclidean, Ward’s) with cuts at 28 (red lines) and 7 clusters (green lines), respectively.

*Nor do they justify the collapse of those categories into seven "meta-clade groupings".*

The seven meta-clade groupings were obtained using the cutree utility in R, applied to the dendrogram of GI50_codebooks_. I agree that the 7 meta-clade groupings (A:G) are not supported as optimal. However, representing the 28 optimal meta-clades as groups of 7 (using the cutree tool on the original 1232 branch dendrogram) simplifies the presentation of results without sacrificing details.

*[11] Using a gradient-based color scheme to label discrete meta-clades and meta-clade groupings in Figure 4 is potentially highly misleading. For one thing, it is extremely difficult to tell if the color boundaries actually align with valid branches in the accompanying dendrogram, especially for the gray-scale meta-clade groupings.* *For visualization, the author might want to consider the Polychrome R package, which can produce a wider variety of distinct colors for displaying discrete classes.*

Heatmap.2 (ggplots package in R) was used to generate the heatmap and adjoining dendrograms. Meta-clades were determined using the cutree utility in R. The colored bar representing meta-clade boundaries is based on using the SidebarColor feature available in Heatmap.2. The image was generated with a single call to heatmap.2. The meta-clade boundaries are aligned as specified by using cutree for 28 meta-clades. The alignment is evident visually from the addition of **Figure 1** (above) to the revised manuscript.

No attempt has been made to mislead anything with the use of a gradient-based color scheme. The spectral representation (blue to red) allows visual separation of the dendrogram branches, such that neighboring branches are also neighbors in the color scheme.

*For visualization, the author might want to consider the Polychrome R package, which can produce a wider variety of distinct colors for displaying discrete classes.*

As an alternative the maxdistcolor utility in MATLAB was used. This scheme allowed each defective gene to have a unique color.

*[12] More importantly, he might want to consider other clustering methods (K-means? PAM?) in light of the fact that the hierarchically defined meta-clades frequently break up into distinct subregions of the SOM display, indicating an incompatibility of clusters between the two methods. As sort-of noted by the author, this disparity is probably caused by changing distance metrics. Both hierarchical clustering and PAM can use the exact same distance metric that was applied when performing SOM clustering. And it would be important to use the same distance metric when applying methods to estimate the number of clusters. Visualizing the data with alternative methods such as t-SNE or UMAP might also provide additional insight into whether one should believe the clustering results.*

The revised manuscript includes the following:

Self-organizing maps (Kohonen) are uniquely positioned in statistical usage. Notable features include its capacity to handle large, noisy datasets with missing values and its non-hierarchical procedures for clustering. The power of SOM’s to map data to a non-hierarchical 2-D hexagonal representation has proven to be a very effective match to the GI50_NCI60_ data. Although pairwise (i.e. hierarchical) associations exist, it is often the case that which pair to select for hierarchical clustering is only marginally different from other alternatives, regardless of the distance metric. The failure to map hierarchical clustering methods (K-means, PAM, AGNES, etc.) directly to a SOM is not unexpected and points more to the limitations of hierarchical methods to match non-hierarchical methods. Thus, switching distance metrics will not likely solve this issue. The submitted manuscript made note of this limitation. However, the strong agreement between the cluster assignments using both methods was noted originally.

The following has also been added to the end of **METHODS: data clustering.**

Noteworthy is the mapping of the 28 cutree clades to discontinuous SOM_DTP_ regions. Ideally, cutree clades might appear as contiguous regions the 2-dimensional SOM_DTP_. However, this is not the case. To obtain contiguous SOM_DTP_ regions, an alternative hierarchical clustering algorithm would need to be used that only combines adjacent dendrogram clades that appear beside each other on SOM_DTP_. Although not pursued here, assigning contiguous SOM regions is an active area of research in dimensionality reduction[11], with specific focus on representing SOMs in one dimension[12]. Many of these efforts use randomized resampling to identify contiguous map regions by consensus. Usually standard hierarchical clustering suffices, and any outlying (noncontiguous) points can be accounted for manually. Towards this end, SOM_DTP_ singletons, appearing as a hierarchical clade that maps to SOM_DTP_ as a node without the same meta-clade neighbors, have been replaced by their neighborhood meta-clade assignments. There are 12 such cases (0.0097=12/1232) for this data set.

An additional consideration for non-contiguous SOM_DTP_ meta-clades may result from the assignment of distances used for clustering (Euclidean for hierarchical clustering and Epanechnikov Function[13] for SOMs). Our choice of the Epanechnikov Function for SOM clustering consistently yielded the lowest SOM_DTP_ quantization errors [14]. However, a more likely explanation for non-contiguous SOM_DTP_ meta-clades involves differences in clustering methodology. SOMs organize data by mapping each cluster to its most similar neighbors (six in the case of hexagonal mapping); whereas hierarchical clustering, as used to obtain heatmap dendrograms, builds each branch of the dendrogram by pairwise associations. The failure to map hierarchical clustering methods directly to contiguous SOM_DTP_ regions is not unexpected and points more to the limitations of hierarchical methods to match non-hierarchical methods, regardless of distance metrics.

*[13] it would be useful to see a version of the original SOM plot (from Figure 2) with the meta-clade boundaries (from Figure 4) superimposed. After all, the original plot indicates the relative distance between neighboring SOM nodes, so it would provide greater support for the clustering if the boundaries tended to run through the yellow areas where neighbors aren't particularly close.*

Thanks and done (See new **Figure 8**).

*[14] From the figure and its caption, it is not clear what we are supposed to learn from Figure 5. My immediate reaction is that ABL1 is not particularly associated with GI50 values in this SOM node. What I get from this plot is that perhaps one should be using a Wilcoxon rank sum test instead of a t-test to associate defective genes with SOM nodes. I have a similar reaction to Figure 6.*

Nonparametric tests (Wilcoxon/Whitney/Mann, Kruskal-Wallis) provide an alternative to parametric tests (Students t-test) when the underlying distribution of the data is either unknown or highly non-normal or ordinal in nature. The data analyzed here represents a strongly normal distribution (p < 0.001) (see figures below) and is appropriate for use of the Students t-test. When suitable, parametric tests offer greater statistical power over nonparametric tests, and are more likely to reveal a true effect in the data. In support of this claim, the case displayed in the original **Figure 5** finds the tumor cell lines with defective *ABL1* to differ significantly (p<6.9e-3) from tumor cells without this defect. Applying a nonparametric rank sum test finds this difference to be not significant. Furthermore, the average response of ABL1 defective genes is 8-fold higher than the average response for non-defective tumor cells. Using a parametric test appears to yield greater statistical power to reveal this response difference when compared to non-parametric tests.

*[15] Figure 7 appears to be somewhat misleading by over-representing the amount of data associated with the CellMiner MOA annotations. While the SOM clusters were created from almost 47000 compounds, the MOA data is only available for 104 compounds, which is only about 0.22% of the total compounds considered. Each of those 104 compounds is then apparently used up to eight times. Is there any statistical significance to any of these "interpretive" assignments?*

The representation of only 0.22% of the screened compounds with FDA-MOA assignments emphasizes the difficulty of finding successful screening hits that lead to FDA approval. A success percentage of 0.22 should be considered exceptional. Extending the SOM_DTP_ nodes to include the top eight SOM_DTP_ projections re-enforces their position on SOM_DTP_. Furthermore, MOA enrichment calculations using additional SOM_DTP_ projections yield more reliable results for then analysis of contingency tables.

*[16] Frankly, I am unclear on exactly how many times Fisher's Exact test was used to associate MOA's with defective genes. Among other things, I have completely lost track of whether these counts are based on SOM nodes, meta-clades, or meta-clade groups. And, once again, there is no sign that any corrections to p-values have been made to account for multiple testing.*

The following section has been added to the revised manuscript.

**METHODS: enrichment of MOA**

Fisher’s exact and chi-square tests are used to identify cases where the SOM_DTP_ projections of defective genes are statistically enriched in co-projections of MOA types. These tests are useful for categorical data that result from classifying objects in two different ways; and are used to determine a statistical measure for the random likelihood of the intersection of each classification. For each defective gene the number of SOM_DTP_ nodes with significant Student’s t-statistics are determined (N_gene_). FDA approved compounds that co-project to N_gene_ determine a unique set of MOA’s associated with each defective gene (MOA_gene_). All FDA compounds that share any MOA_gene_ are collected (N_FDA_); where the 10^th^ best FDA projections are included in the count. Extending FDA projections beyond only the best node achieves two goals. First, it establishes SOM_DTP_ regions rather than individual nodes for MOA classification. Second, increasing the numbers in the contingency table extends significance testing to include Fisher’s exact and the chi-square testing. The contingency table entries become; p_11_=intersection(N_gene_,N_FDA_), p_12_=N_gene_ – p11, p_21_=N_FDA_ – p_22_ and p_22_=p_11_ by default to conserve equal row and column sums.

**Figure 6** illustrates a sample result of the steps for calculating the Fisher’s exact statistic using *ABL1*. **Panel A** (reproduced from **Figure 4**) displays the significant SOM_DTP_ nodes for defective *ABL1*, where N_gene_=48. Collecting the MOA’s for the 11 FDA compounds co-projected to N_gene_ finds 6 MOAs (Apo, Ho, HSP90, NonCan, PK and BCR-ABL). **Panel B** in **Figure 6** displays the top 10^th^ percentile of all FDA compounds sharing any one of these 6 MOAs, to yield N_FDA_=189. Completing the contingency table with their intersection (22, results in a Fisher’s exact score of 1.958262e-09 (logpval(-20.051208)).

**Figure 6: Panel A** displays the significant SOM_DTP_ nodes for *ABL1* (N_gene_=48). Eleven FDA compounds are co-projected to N_gene_; yielding 6 MOAs. The SOM_DTP_ in **Panel B** displays the top 10^th^ percentile of projections for FDA compounds sharing these MOAs (N_FDA_=189). The intersection of N_gene_ and N_FDA_ = 22, yielding a Fishers exact p-value of 1.958262e-09, log(p-value= -20.05).

The MOA enrichment analysis was completed on the 7 meta-clade groups (**A-G**). Applying contingency tests to these groups, rather that the 28 meta-clades, increased the numbers in the contingency tables and by doing so supported the addition of a chi-square statistic to complement the Fisher’s exact results.

*[17] Beginning on page 17 of the document for reviewers, the author drifts from presenting results to discussing their interpretation. It would be easier for the reader if these parts of the manuscript remained more clearly separated.*

Thanks again. This comment was the basis for completely reorganizing the revised manuscript. Hopefully this revision allows clearer separation of **DATA**, **METHODS** and **RESULTS**.

*[18] It would be nice if the font sizes in the numerous tables were large enough to be readable.*

In addition to increasing font sizes in these Figures, the tabular results are now included as multiple sheets within a master excel file included in the **Appendix.**

*Journal requirements:*

*When submitting your revision, we need you to address these additional requirements.*

*1. Please ensure that your manuscript meets PLOS ONE's style requirements, including those for file naming. The PLOS ONE style templates can be found at*

*https://journals.plos.org/plosone/s/file?id=wjVg/PLOSOne_formatting_sample_main_body.pdf and*

*https://journals.plos.org/plosone/s/file?id=ba62/PLOSOne_formatting_sample_title_authors_affiliations.pdf*

*2. Thank you for stating in your Funding Statement:*

*"This project was funded in whole or in part with Federal funds from the National Cancer Institute, National Institutes of Health, under Contracts No. HHSN261200800001E and HHSN261201700007I."*

*Please provide an amended statement that declares *all* the funding or sources of support (whether external or internal to your organization) received during this study, as detailed online in our guide for authors at http://journals.plos.org/plosone/s/submit-now. Please also include the statement* *“**There was no additional external funding received for this study.” in your updated Funding Statement.*

*Please include your amended Funding Statement within your cover letter. We will change the online submission form on your behalf.*

Thanks. The Funding Statement now includes:

There was no additional external funding received for this study.

*3. Please include captions for your Supporting Information files at the end of your manuscript, and update any in-text citations to match accordingly. Please see our Supporting Information guidelines for more information: http://journals.plos.org/plosone/s/supporting-information.*

*[Note: HTML markup is below. Please do not edit.]*

*Comments to the Author*

*1. Is the manuscript technically sound, and do the data support the conclusions?*

*The manuscript must describe a technically sound piece of scientific research with data that supports the conclusions. Experiments must have been conducted rigorously, with appropriate controls, replication, and sample sizes. The conclusions must be drawn appropriately based on the data presented.*

*Reviewer #1: Partly*

*Reviewer #2: Yes*

*________________________________________*

*2. Has the statistical analysis been performed appropriately and rigorously?*

*Reviewer #1: No*

*Reviewer #2: I Don't Know*

*________________________________________*

*3. Have the authors made all data underlying the findings in their manuscript fully available?*

*The PLOS Data policy requires authors to make all data underlying the findings described in their manuscript fully available without restriction, with rare exception (please refer to the Data Availability Statement in the manuscript PDF file). The data should be provided as part of the manuscript or its supporting information, or deposited to a public repository. For example, in addition to summary statistics, the data points behind means, medians and variance measures should be available. If there are restrictions on publicly sharing data—e.g. participant privacy or use of data from a third party—those must be specified.*

*Reviewer #1: No*

*Reviewer #2: No*

*________________________________________*

*4. Is the manuscript presented in an intelligible fashion and written in standard English?*

*PLOS ONE does not copyedit accepted manuscripts, so the language in submitted articles must be clear, correct, and unambiguous. Any typographical or grammatical errors should be corrected at revision, so please note any specific errors here.*

*Reviewer #1: Yes*

*Reviewer #2: Yes*

*________________________________________*

*5. Review Comments to the Author*

*Please use the space provided to explain your answers to the questions above. You may also include additional comments for the author, including concerns about dual publication, research ethics, or publication ethics. (Please upload your review as an attachment if it exceeds 20,000 characters)*

*Reviewer #1: See attached file.*

*Reviewer #2: In the manuscript, the author presents work to integrate chemosensitivity of cancer cell lines with their genomic defects and drug mechanisms of FDA-approved agents. The work is an important and useful study, particularly as medical oncology moves towards a targeted, individualized approach to treat each individual’s cancer with a personalized drug combination. Drug re-purposing and drug combinations, along with treatment sequence, all play into this, and the more information we have about how chemosensitivity, genomic alterations and drug action link together, it may be possible to discover how drugs synergize to create a lethal combination to treat many cancers. Knowing the genetic vulnerabilities of an individual’s tumor type is becoming more possible and cost-efficient with the advances in sequencing technologies.*

*The author extends work by Ikediobi et al. by the inclusion of more screened compounds (~53k vs ~8k) and a larger set of gene mutations (N=365 vs N=24). A novel analysis of self-organizing-maps (SOMs) integrated data from three different sources (NCI60, cBioPortal and CellMiner) and derived links among tumor cell chemosensitivity, genetically defective genes and mechanisms of action (MOA).*

*While this study is limited to the analysis of data from cancer cell lines, the results are applicable and could be beneficial for pre-clinical studies. This is pointed out in the Discussion as cell lines are not fully representative of human tumors.*

*Overall the author could improve the structure of the manuscript for readability. For example, the introduction is very brief and ends abruptly with the bullet point list. This should be expanded.*

I agree. The structure of the original manuscript has been significantly altered with the aim of separating the DATA, METHODS and RESULTS sections and enhancing readability. With respect to the specific suggestion to extend the introduction the following has been included after the bullet points.

Efforts to develop links between pre-clinical tumor cell screening data, genomic defects and drug mechanisms may contribute to advances in small-molecule cancer therapies. An important element of these efforts requires more informed interpretations of small-molecule screening results in the context of genomic profiles and drug action. These associations may yield undiscovered opportunities for drug re-purposing and new applications of gene mutations towards personalized medicine.

*The Methods/Data focuses on describing the boxes in Figure 1. A better approach might be to break this into headings, and then refer to Figure 1, Box A, rather than have Box A as the heading. This is particularly the case when the author goes on to refer to Box B – E. Descriptive headings would be much more useful to the reader.*

The original Figure 1 has been eliminated in favor of subheadings for the sections covering the DATA, METHODS and RESULTS. This suggestion has improved readability and avoids the problems associated with the confusion of intermingling these sections.

*The figure legends should have titles – what is the result of the figure? Then a description of each panel should follow. In several figures, it would be helpful to increase the size and include panel letters. While many figures have multiple panels, they are referred to as left/right or upper/lower etc. Panel letters would improve clarity. Many figure legends are too brief. Expansion of the figure legends to fully explain the figures would improve readability of the manuscript.*

All Figures now have legends that provide a description of content. Expanded coverage exists in the text referring to each figure. Panels within each figure are now labelled with letters (**A, B**, etc) rather than position.

*For someone not directly involved in the field, it would be helpful to define terms, i.e. DTP, NSC, etc. This is also true for gene/proteins, like KRAS, etc., and cancer types (TNBC).*

Thanks. An ABBREVIATOINS section is now included. HUGO names for genes are used and where appropriate an expanded description for each protein is provided.

*There is a misuse of verb tense throughout the manuscript. For example, “Prior analyses find…” – this should be “found”. Many parts are written as if the work is proposed, not completed.*

My apologies. I believe verb tenses are now correct.

*Gene names should be italicized (not bold); human proteins in all capitals, non-italicized. This is standard convention and would improve the clarity when moving between defective genes and their resultant proteins and protein activities.*

Thanks.

*In some cases, work of others is referred to without the reference, i.e. in Appendix Figure 2 and Holbeck et al. (in the description for Box C).*

Corrected (see above also).

*Figure 2 – it is very difficult to see the drug names that are in white against the colored SOMDTP projections. The MOAs for the drug lists should be specified on the figure. This is listed in the text, but better labelling of the figure would improve clarity.*

The original Figure 2 has been redone and now appears in the **RESULTS: SOM_DTP_** section as **Figure 8**. The SOM_DTP_ is now displayed with the 28-meta-clade boundaries and the 7 meta-cade groups. FDA compound names for each meta-clade now appear as a table (per Reviewer 1’s suggestion) **Panel C** in **Figure 8**. MOA’s for FDA compounds appear as a sheet within an excel file included in the Appendix (master_appendix sheet appendix_table III). Inclusion of new tables adds details that have not yet been brought into the manuscript. **Panels C** and **D** of **Figure 8** are provided below.

*Figure 3 – the x-axis on each plot is very difficult to see. How is the inflection point of gap statistic and within sum of squares method calculated? Was this a calculation or interpreted from the curves? With this and other figures, more details and primary data used to do these analyses should be included.*

I agree. The revised manuscript provides an expanded coverage of the methods used to determine the optimal number of clusters. The following Figure has been included.

**Figure 1**. Results for selecting optimal cluster size using the **elbow** method (**Panel A**), **silhouette** method (**Panel B**) and the **gap**_**statistic** method (**Panel C**). Selection for the **elbow** method is based on the largest local derivative of the within groups sum of squares (**Panel A** triangles), the maximum silhouette width (**Panel B**) and the first non-negative value for Gap(k)-(Gap(k + 1)−sd_k+1_) (**Panel C** triangles). These results indicate an optimal number of clusters in the 26-28 range. **Panel D** displays the GI50_codebook_ dendrogram (Euclidean, Ward’s) with cuts at 28 (red lines) and 7 clusters (green lines), respectively.

*Pg 13 – surely there is a reference for nonlinear dimension reduction that is not Wikipedia?*

My mistake.

20.	Lawrence ND. A Unifying Probabilistic Perspective for Spectral Dimensionality Reduction: Insights and New Models2010 October 01, 2010:[arXiv:1010.4830 p.]. Available from: <https://ui.adsabs.harvard.edu/abs/2010arXiv1010.4830L>.

21.	Gansner ER, Hu Y, Kobourov S, editors. GMap: Visualizing graphs and clusters as maps. 2010 IEEE Pacific Visualization Symposium (PacificVis); 2010 2-5 March 2010.

*Pg 14/Figure 5 – it would be helpful to have an indication/description of what the cell lines are. This is done for Figure 6; however, it should read, “Here there are 12 tumor cell lines…” not tumor cells.*

The tumor cell lines are now included in the legend of **Figure 1**. I have replaced reference to tumor cells with tumor cell lines.

*The clinical significance of the results of the study could be expanded. What will be the next steps using the results obtained?*

The following is now included in the **DISCUSSION**.

The results described here may be applied to future pre-clinical studies. Notably there are exploitable instances of enhanced chemosensitivity of compound MOA’s for a few defective genes. Specifically, there is support for synthetically lethal defective genes as contributing to chemosensitivity. Defective genes exist withing the NCI60 as doublets, triplets, quartets, etc., and a subset of these genes are associated with tumor cell lines that exhibit chemosensitivity. Exploiting chemosensitive SOM_DTP_ nodes associated with tumor cell lines having more than one defective gene, that are also associated with numerous screened compounds, may identify additional synthetic lethal strategies. The notion of targeting parallel pathways can be extend beyond synthetically lethal genes. Combining agents with enhanced chemosensitivity against one defective gene, and its related cellular pathways, with other agents showing enhanced chemosensitivity towards other defective genes in alternative pathways, may enhance the efficacy of each agent. For example, each SOM_DTP_ node with significant chemosensitivity for one defective gene includes many NSCs with similar GI50_NCI60_ responses, inclusive of FDA compounds. Combinations of NSCs from SOM_DTP_ nodes also exhibiting differential chemosensitivity for one or more defective gene in parallel pathways may be considered for experimental testing. The goal would be to identify combinations of NSCs that separately target parallel cellular pathways to determine whether their combination would enhance individual efficacies. The bioinformatic analysis described herein may provide clues for experimental pre-clinical testing of possible drug combinations.

*For Appendix Figures 3 and 4 – is the result of the defective PI3KR1 and IGFR1 surprising?*

The result for *PI3KR1* provides support for the earlier report of Whyte and Holbeck[15]. The *IGFR1* result is surprising for 2 reasons. First, defective *IGFR1*, to my knowledge, has not been noted previously to be a part of the genetic makeup of the NCI60 leukemia cell line. Second, the importance of defective *IGFR1* in treatment selection has been reported in clinical settings. Relating the NCI60 results to these clinical settings adds utility to these preclinical results.

*The headings for Table II could be improved to describe what will follow. More detail could be added to improve readability.*

The **RESULTS** section has been modified to report each of the 7 meta-clade groups (**A-G**). These results appear as panels **A-D** in the figures associated with each group. The font sizes have been increased for readability. A master excel file has been included in the **Appendix** to list the tables displayed in **Panel C** for these figures.

*Figures 9 – 14 – each right panel displays partial SOMDTP meta-clades, which are shown fully in Figure 4. It would be helpful to refer back to Figure 4 or superimpose the full SOMDTP for context.*

Thanks. The legends for these figures now include a reference back to the original SOM_DTP_. For example, the legend to **Figure 9** now includes;

The SOM_DTP_ region displayed in **Panel B** represents the boundary for meta-clades 1 through 6 (see the white border in **Figure 8 Panel A**).

*The author refers to ‘defective’ genes throughout; however, it would be useful to point out that mutations, copy number alterations and/or fusion/splice changes can enable and ‘over-activate’ or enhance gene function. Defective implies lack of function, which is not the case with some of the genes mentioned, particularly in a cancer context. Some specific examples do not fall into this (i.e. p. 27 V600E activating mutation of BRAF); however, the author should clearly delineate the difference between defective/lack-of-function versus altered/gain-of-function.*

Thank you for the comment. The paragraph on new page 3 has been modified as follows:

Second, genetic data is obtained from the cBioPortal database (<https://www.cbioportal.org/>) [16, 17]. A total of 368 altered genes are listed for the NCI60; with either a mutation (MUT), copy number alteration (CNA) or fusion/splice (FUSION). These genomic changes are grouped, so that a gene alteration due to any or all types of variations will be designated as genetically defective. In this context, a defective gene indicates only a modification from the wild-type allele. Genes designated as defective genes can have wide-ranging effects including gain-of and/or loss-of gene function.

*________________________________________*

*6. PLOS authors have the option to publish the peer review history of their article (what does this mean?). If published, this will include your full peer review and any attached files.*

*If you choose “no”, your identity will remain anonymous but your review may still be made public.*

*Do you want your identity to be public for this peer review? For information about this choice, including consent withdrawal, please see our Privacy Policy.*

*Reviewer #1: Yes: Kevin R Coombes*

*Reviewer #2: No*

*[NOTE: If reviewer comments were submitted as an attachment file, they will be attached to this email and accessible via the submission site. Please log into your account, locate the manuscript record, and check for the action link "View Attachments". If this link does not appear, there are no attachment files.]*

*While revising your submission, please upload your figure files to the Preflight Analysis and Conversion Engine (PACE) digital diagnostic tool, https://pacev2.apexcovantage.com/. PACE helps ensure that figures meet PLOS requirements. To use PACE, you must first register as a user. Registration is free. Then, login and navigate to the UPLOAD tab, where you will find detailed instructions on how to use the tool. If you encounter any issues or have any questions when using PACE, please email PLOS at figures@plos.org. Please note that Supporting Information files do not need this step.*

*________________________________________*

*In compliance with data protection regulations, you may request that we remove your personal registration details at any time. (Remove my information/details). Please contact the publication office if you have any questions.*

1.	Vesanto J, Alhoniemi E. Clustering of the self-organizing map. IEEE Trans Neural Netw. 2000;11(3):586-600. Epub 2008/02/06. doi: 10.1109/72.846731. PubMed PMID: 18249787.

2.	Kohonen T. Essentials of the self-organizing map. Neural Netw. 2013;37:52-65. Epub 2012/10/17. doi: 10.1016/j.neunet.2012.09.018. PubMed PMID: 23067803.

3.	Diaconis P, Efron B. Computer-intensive methods in statistics. Sci Am; (United States). 1983:Medium: X; Size: Pages: 116-30.

4.	Langham J. Ranking small molecules by how much they preferentially inhibit the growth of cancer cell lines with either BRAF or KRAS oncogene mutations. PeerJ Preprints; 2014.

5.	Jafari M, Ansari-Pour N. Why, When and How to Adjust Your P Values? Cell J. 2019;20(4):604-7. Epub 2018/08/21. doi: 10.22074/cellj.2019.5992. PubMed PMID: 30124010; PubMed Central PMCID: PMCPMC6099145.

6.	Kohonen T. The self-organizing map. Proceedings of the IEEE. 1990;78(9):1464-80. doi: 10.1109/5.58325.

7.	Benjamini Y, Hochberg Y. Controlling the False Discovery Rate: A Practical and Powerful Approach to Multiple Testing. Journal of the Royal Statistical Society: Series B (Methodological). 1995;57(1):289-300. doi: <https://doi.org/10.1111/j.2517-6161.1995.tb02031.x>.

8.	Efron B. A 250-year argument: Belief, behavior, and the bootstrap. Bulletin of the American Mathematical Society. 2012;50(1):129-46. doi: 10.1090/s0273-0979-2012-01374-5.

9.	Tibshirani R, Walther G, Hastie T. Estimating the number of clusters in a data set via the gap statistic. J Roy Stat Soc B. 2001;63:411-23. doi: Doi 10.1111/1467-9868.00293. PubMed PMID: WOS:000168837200013.

10.	Bodenhofer U, Kothmeier A, Hochreiter S. APCluster: an R package for affinity propagation clustering. Bioinformatics. 2011;27(17):2463-4. Epub 2011/07/09. doi: 10.1093/bioinformatics/btr406. PubMed PMID: 21737437.

11.	Lawrence ND. A Unifying Probabilistic Perspective for Spectral Dimensionality Reduction: Insights and New Models2010 October 01, 2010:[arXiv:1010.4830 p.]. Available from: <https://ui.adsabs.harvard.edu/abs/2010arXiv1010.4830L>.

12.	Gansner ER, Hu Y, Kobourov S, editors. GMap: Visualizing graphs and clusters as maps. 2010 IEEE Pacific Visualization Symposium (PacificVis); 2010 2-5 March 2010.

13.	Epanechnikov VA. Non-Parametric Estimation of a Multivariate Probability Density. 1969;14(1):153-8. doi: 10.1137/1114019.

14.	Rabow AA, Shoemaker RH, Sausville EA, Covell DG. Mining the National Cancer Institute's tumor-screening database: identification of compounds with similar cellular activities. J Med Chem. 2002;45(4):818-40. Epub 2002/02/08. doi: 10.1021/jm010385b. PubMed PMID: 11831894.

15.	Whyte DB, Holbeck SL. Correlation of PIK3Ca mutations with gene expression and drug sensitivity in NCI-60 cell lines. Biochem Biophys Res Commun. 2006;340(2):469-75. Epub 2005/12/27. doi: 10.1016/j.bbrc.2005.12.025. PubMed PMID: 16376301.

16.	Cerami E, Gao J, Dogrusoz U, Gross BE, Sumer SO, Aksoy BA, et al. The cBio cancer genomics portal: an open platform for exploring multidimensional cancer genomics data. Cancer Discov. 2012;2(5):401-4. Epub 2012/05/17. doi: 10.1158/2159-8290.CD-12-0095. PubMed PMID: 22588877; PubMed Central PMCID: PMCPMC3956037.

17.	Gao J, Aksoy BA, Dogrusoz U, Dresdner G, Gross B, Sumer SO, et al. Integrative analysis of complex cancer genomics and clinical profiles using the cBioPortal. Sci Signal. 2013;6(269):pl1. Epub 2013/04/04. doi: 10.1126/scisignal.2004088. PubMed PMID: 23550210; PubMed Central PMCID: PMCPMC4160307.
